# Supplementary material for: Predicting yield of individual field-grown rapeseed plants from rosette-stage leaf gene expression
Source: PLoS Comput Biol. 2023 May 30;19(5):e1011161. doi: 10.1371/journal.pcbi.1011161 (PMC10256231; doi:10.1371/journal.pcbi.1011161)
Supplement: S1 Fig — (PDF) [file pcbi.1011161.s001.pdf]

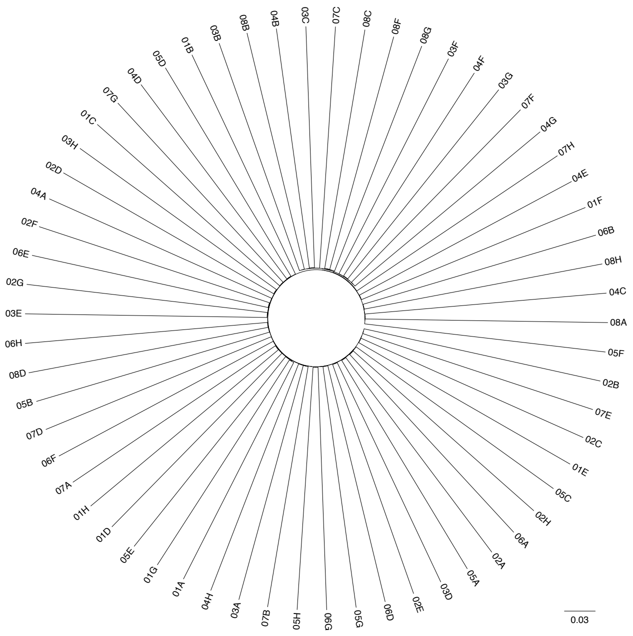


**S1 Fig. SNP analysis of individual plants.** Neighbor-joining tree for the individual plants based on biallelic SNPs in the RNA-seq data. Branch lengths are proportional to 1 – IBS (identity by state).
