# Supplementary material for: Predicting yield of individual field-grown rapeseed plants from rosette-stage leaf gene expression
Source: PLoS Comput Biol. 2023 May 30;19(5):e1011161. doi: 10.1371/journal.pcbi.1011161 (PMC10256231; doi:10.1371/journal.pcbi.1011161)
Supplement: S2 Fig — (PDF) [file pcbi.1011161.s002.pdf]

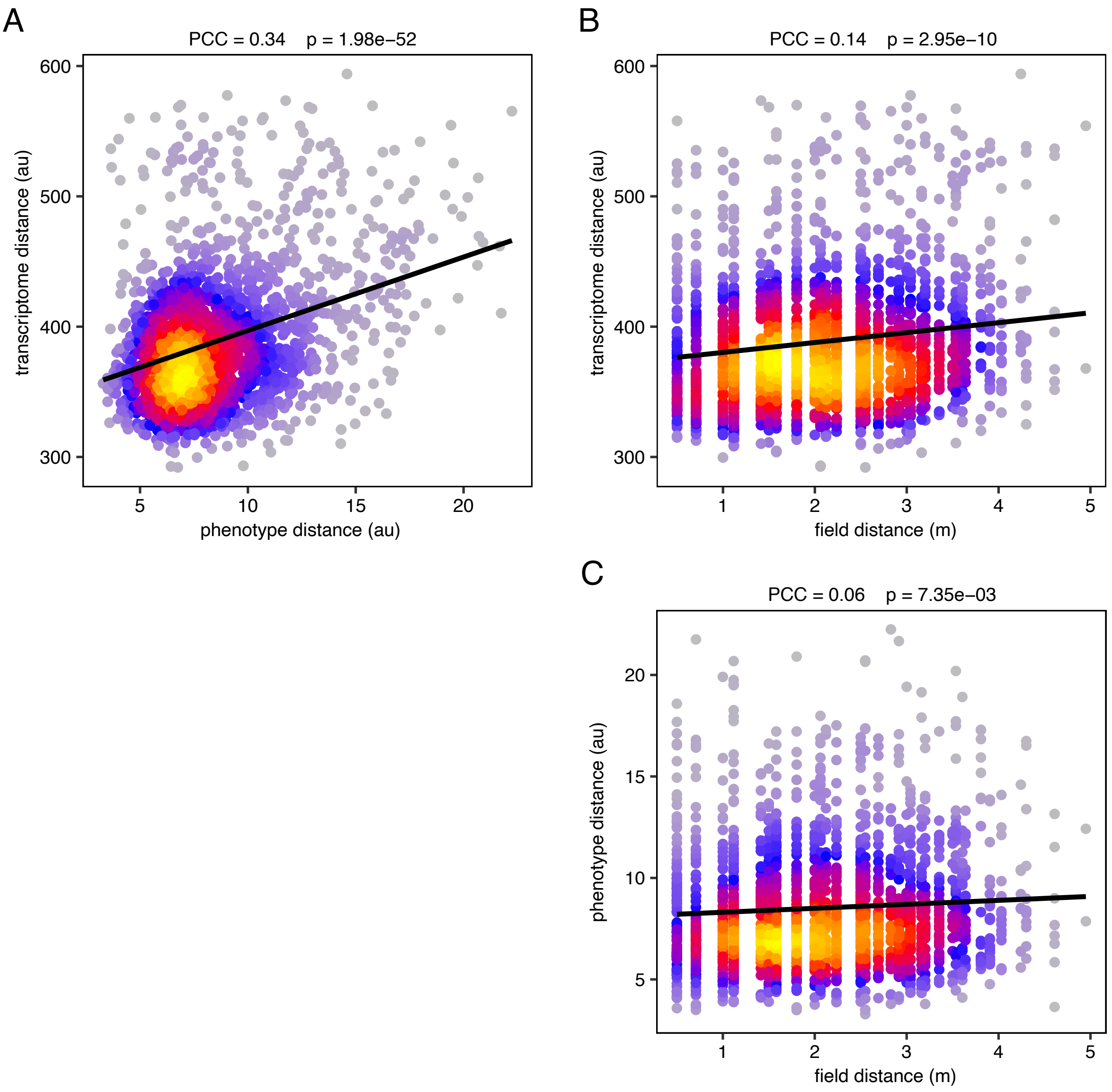

S2 Fig. Correlations of pairwise distances between plants across different omics layers. In panel **A**, the Euclidean distance between plants in transcriptome space is plotted against their Euclidean distance in phenotype space. Panels **B** and **C** similarly compare the Euclidean distance between plants in transcriptome and phenotype space, respectively, with the physical distance between plants in the field. Each dot represents a pair of plants, colors reflect dot density (yellow = high density, grey = low density). Gene expression and phenotype profiles across plants were z-scored before computing distances. Missing phenotype values were imputed using Bayesian PCA (see Variance analysis section in the Methods). The black lines on the panels are linear regression lines. The Pearson correlation coefficient and associated *p*-value are shown above each panel. Distances between plants in transcriptome and phenotype space exhibit substantial correlation. The correlation between either of these distances and the physical distance between plants in the field is weak but statistically significant.
