# Supplementary material for: Predicting yield of individual field-grown rapeseed plants from rosette-stage leaf gene expression
Source: PLoS Comput Biol. 2023 May 30;19(5):e1011161. doi: 10.1371/journal.pcbi.1011161 (PMC10256231; doi:10.1371/journal.pcbi.1011161)
Supplement: S3 Fig — (PDF) [file pcbi.1011161.s003.pdf]

**S3 Fig. Phenotype field plots.** Each plot displays the variation of a phenotype over the field. Shown on top of each plot are the Moran's I value of the phenotype (calculated using a queen contiguity-based spatial weight matrix), the expected Moran's I (by resampling) and the associated *p*-value and *q*-value (computed using the Benjamini-Hochberg method over all phenotypes). The table below orders phenotypes by increasing *p*-value, the page and panel numbers of each plot are given in the 'page' and 'panel' columns, respectively.

| phenotype | Moran's I | p-value | q-value | page | panel |
|---|---|---|---|---|---|
| root system width | -0.201762 | 0.00059 | 0.024190 | 6 | D |
| rosette area (42 DAS) | -0.155723 | 0.00977 | 0.193930 | 2 | A |
| plant height (278 DAS) | 0.150075 | 0.01419 | 0.193930 | 8 | D |
| seed weight stem 1/dry weight stem 1 | -0.146537 | 0.02387 | 0.244668 | 6 | F |
| total seed weight/shoot dry weight | -0.132836 | 0.03028 | 0.248296 | 6 | E |
| branch count stem 1/length stem 1 | -0.125956 | 0.04515 | 0.281143 | 4 | F |
| rosette lesions (74 DAS) | 0.103692 | 0.04800 | 0.281143 | 2 | E |
| leaf 8 lesions (76 DAS) | 0.065153 | 0.11645 | 0.460654 | 3 | C |
| siliques per branch | 0.063923 | 0.11864 | 0.460654 | 6 | A |
| taproot length | 0.062792 | 0.12300 | 0.460654 | 6 | C |
| leaf 6 length (74 DAS) | 0.061927 | 0.12359 | 0.460654 | 2 | B |
| siliques per branch stem 1 | 0.049414 | 0.16069 | 0.494193 | 6 | B |
| max shoot growth rate | 0.045331 | 0.18007 | 0.494193 | 8 | A |
| silique count stem 1 | 0.039144 | 0.19681 | 0.494193 | 5 | F |
| leaf 8 chlorophyll content (81 DAS) | 0.033513 | 0.22412 | 0.494193 | 3 | D |
| total branch count | -0.068774 | 0.23080 | 0.494193 | 4 | C |
| dry weight stem 1 | 0.028904 | 0.23783 | 0.494193 | 5 | B |
| dry weight stem 1 (w/o seeds) | 0.026155 | 0.24807 | 0.494193 | 5 | D |
| leaf 8 area (81 DAS) | 0.021102 | 0.27089 | 0.494193 | 3 | E |
| seed weight stem 1 | 0.015635 | 0.29884 | 0.494193 | 7 | F |
| seed count stem 1 | 0.013265 | 0.30896 | 0.494193 | 7 | B |
| leaf 8 fresh weight (81 DAS) | 0.013253 | 0.31319 | 0.494193 | 4 | A |
| end of shoot growth | -0.051703 | 0.31778 | 0.494193 | 8 | B |
| leaf 8 length (81 DAS) | 0.009660 | 0.32878 | 0.494193 | 3 | F |
| seeds per silique | -0.041364 | 0.35952 | 0.494193 | 7 | C |
| branch count stem 1 | -0.043333 | 0.36680 | 0.494193 | 4 | D |
| time of max shoot growth | -0.039322 | 0.38448 | 0.494193 | 8 | C |
| total shoot dry weight (w/o seeds) | -0.005834 | 0.41398 | 0.494193 | 5 | C |
| leaf 8 width (81 DAS) | -0.007272 | 0.42161 | 0.494193 | 4 | B |
| leaf 8 width (76 DAS) | -0.008046 | 0.42658 | 0.494193 | 3 | B |
| stem count | -0.032520 | 0.43216 | 0.494193 | 8 | E |
| total shoot dry weight | -0.010028 | 0.43615 | 0.494193 | 5 | A |
| leaf 6 width (74 DAS) | -0.012587 | 0.45033 | 0.494193 | 2 | C |
| total seed count | -0.028611 | 0.45423 | 0.494193 | 7 | A |
| leaf count (74 DAS) | -0.013497 | 0.46013 | 0.494193 | 2 | D |
| seeds per silique stem 1 | -0.014360 | 0.46461 | 0.494193 | 7 | D |
| leaf 8 length (76 DAS) | -0.015821 | 0.47168 | 0.494193 | 3 | A |
| total seed weight | -0.024872 | 0.47461 | 0.494193 | 7 | E |
| leaf 6 lesions (74 DAS) | -0.023718 | 0.48178 | 0.494193 | 2 | F |
| total silique count | -0.018128 | 0.48214 | 0.494193 | 5 | E |
| branches per stem | -0.020223 | 0.49691 | 0.496910 | 4 | E |

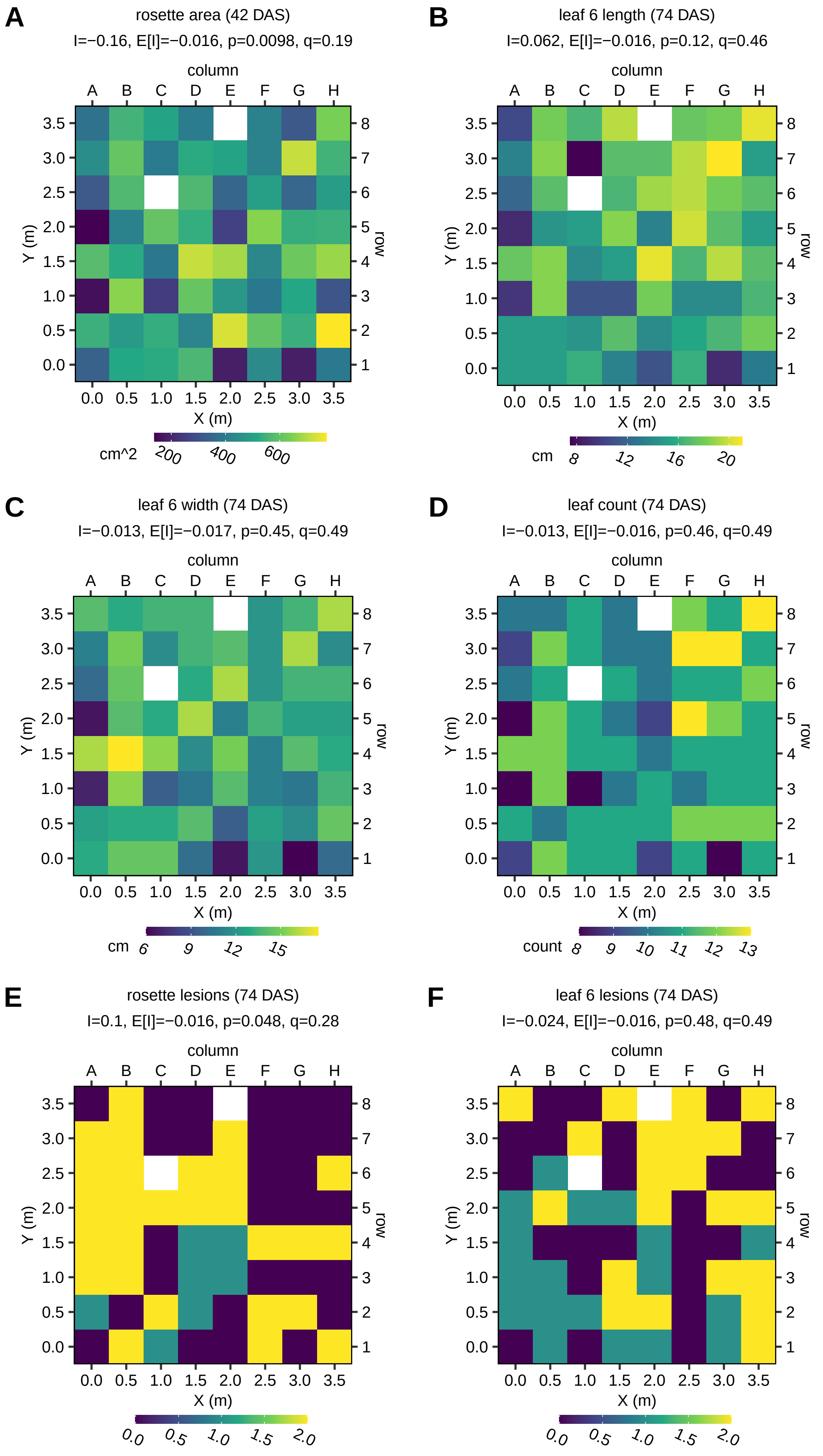
A
rosette area (42 DAS)
I=−0.16, E[I]=−0.016, p=0.0098, q=0.19
cm^2
B
leaf 6 length (74 DAS)
I=0.062, E[I]=−0.016, p=0.12, q=0.46
cm
C
leaf 6 width (74 DAS)
I=−0.013, E[I]=−0.017, p=0.45, q=0.49
cm
D
leaf count (74 DAS)
I=−0.013, E[I]=−0.016, p=0.46, q=0.49
count
E
rosette lesions (74 DAS)
I=0.1, E[I]=−0.016, p=0.048, q=0.28
F
leaf 6 lesions (74 DAS)
I=−0.024, E[I]=−0.016, p=0.48, q=0.49
column
row
X (m)
Y (m)

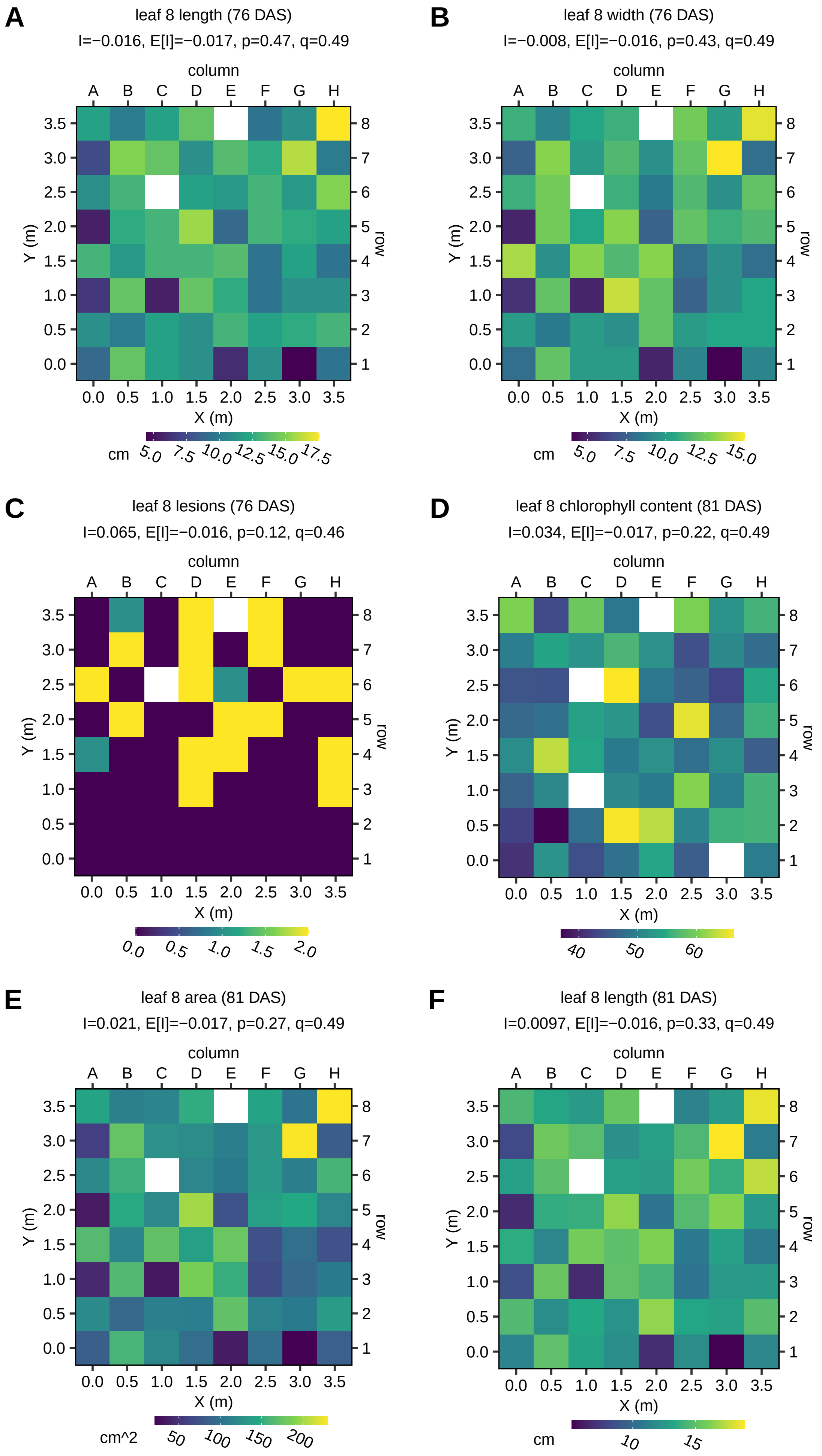
A
leaf 8 length (76 DAS)
I=−0.016, E[I]=−0.017, p=0.47, q=0.49
column
A B C D E F G H
3.5 3.0 2.5 2.0 1.5 1.0 0.5 0.0
Y (m)
8 7 6 5 4 3 2 1
row
0.0 0.5 1.0 1.5 2.0 2.5 3.0 3.5
X (m)
cm 5.0 7.5 10.0 12.5 15.0 17.5
B
leaf 8 width (76 DAS)
I=−0.008, E[I]=−0.016, p=0.43, q=0.49
cm 5.0 7.5 10.0 12.5 15.0
C
leaf 8 lesions (76 DAS)
I=0.065, E[I]=−0.016, p=0.12, q=0.46
0.0 0.5 1.0 1.5 2.0
D
leaf 8 chlorophyll content (81 DAS)
I=0.034, E[I]=−0.017, p=0.22, q=0.49
40 50 60
E
leaf 8 area (81 DAS)
I=0.021, E[I]=−0.017, p=0.27, q=0.49
cm^2 50 100 150 200
F
leaf 8 length (81 DAS)
I=0.0097, E[I]=−0.016, p=0.33, q=0.49
cm 10 15

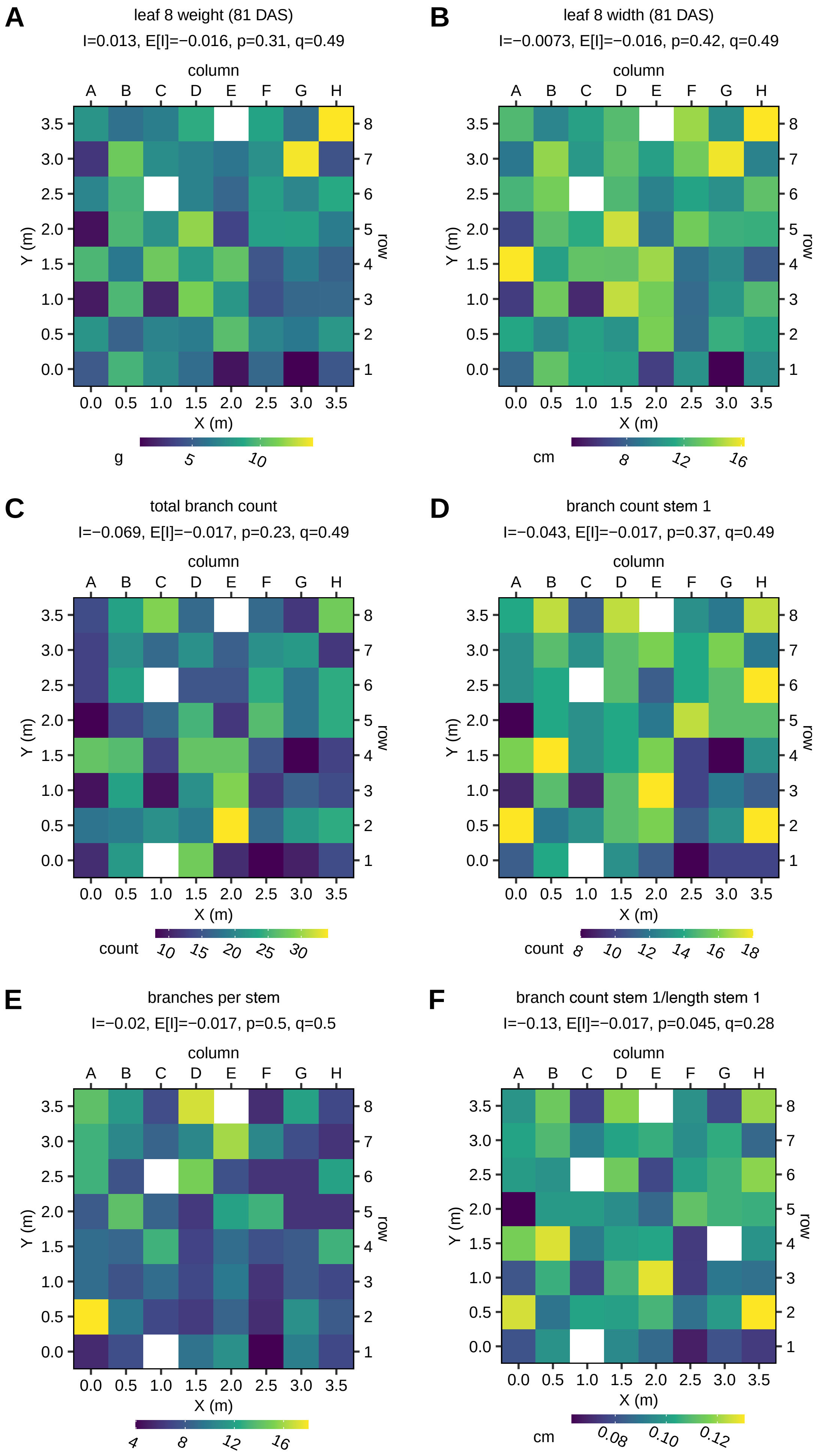
A
leaf 8 weight (81 DAS)
I=0.013, E[I]=−0.016, p=0.31, q=0.49
B
leaf 8 width (81 DAS)
I=−0.0073, E[I]=−0.016, p=0.42, q=0.49
C
total branch count
I=−0.069, E[I]=−0.017, p=0.23, q=0.49
D
branch count stem 1
I=−0.043, E[I]=−0.017, p=0.37, q=0.49
E
branches per stem
I=−0.02, E[I]=−0.017, p=0.5, q=0.5
F
branch count stem 1/length stem 1
I=−0.13, E[I]=−0.017, p=0.045, q=0.28
column
A B C D E F G H
row
1 2 3 4 5 6 7 8
Y (m)
X (m)
0.0 0.5 1.0 1.5 2.0 2.5 3.0 3.5
g 5 10
cm 8 12 16
count 10 15 20 25 30
count 8 10 12 14 16 18
4 8 12 16
cm 0.08 0.10 0.12

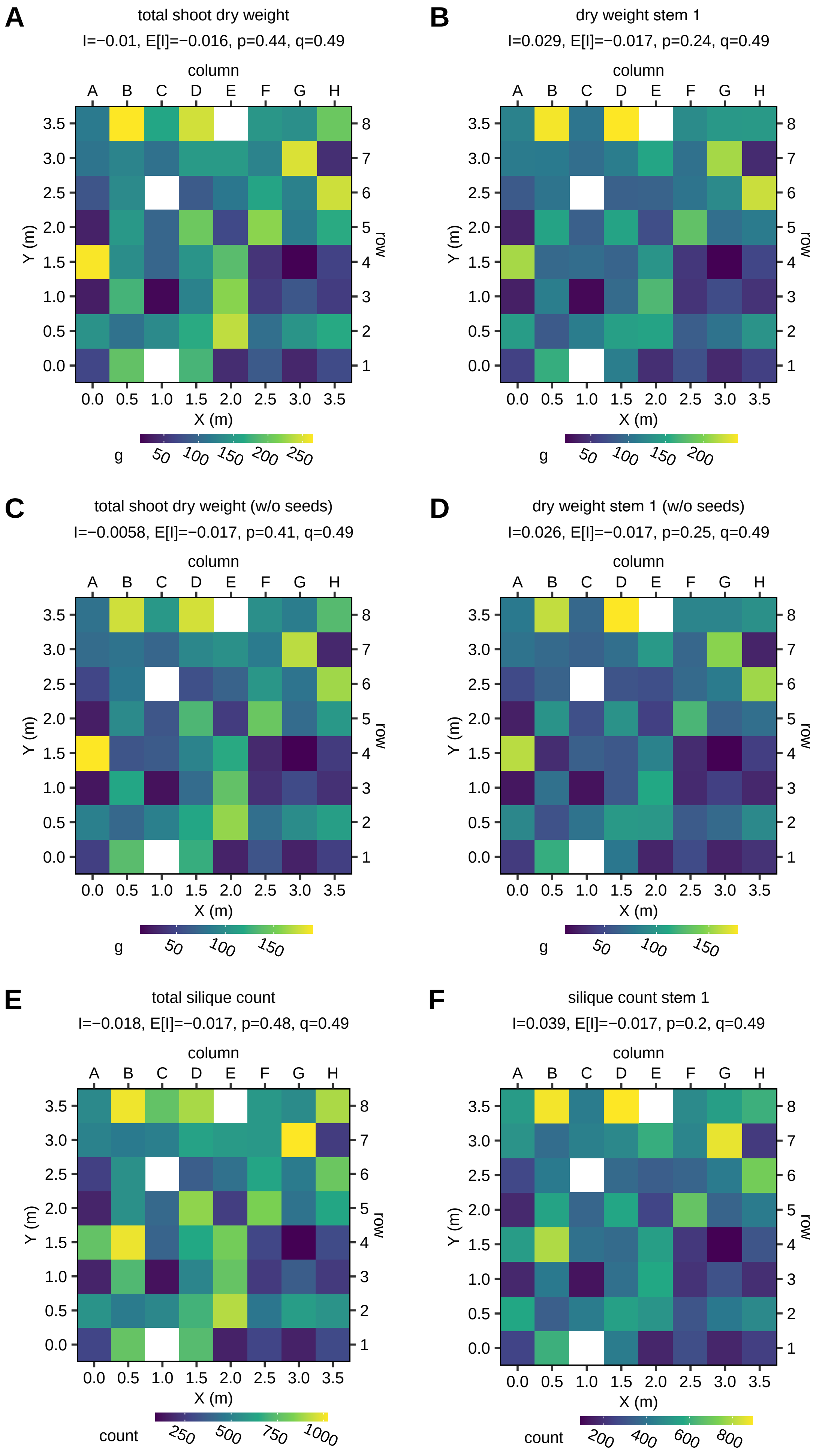
A
total shoot dry weight
I=−0.01, E[I]=−0.016, p=0.44, q=0.49
B
dry weight stem 1
I=0.029, E[I]=−0.017, p=0.24, q=0.49
C
total shoot dry weight (w/o seeds)
I=−0.0058, E[I]=−0.017, p=0.41, q=0.49
D
dry weight stem 1 (w/o seeds)
I=0.026, E[I]=−0.017, p=0.25, q=0.49
E
total silique count
I=−0.018, E[I]=−0.017, p=0.48, q=0.49
F
silique count stem 1
I=0.039, E[I]=−0.017, p=0.2, q=0.49
column
A B C D E F G H
Y (m)
X (m)
row
g
count

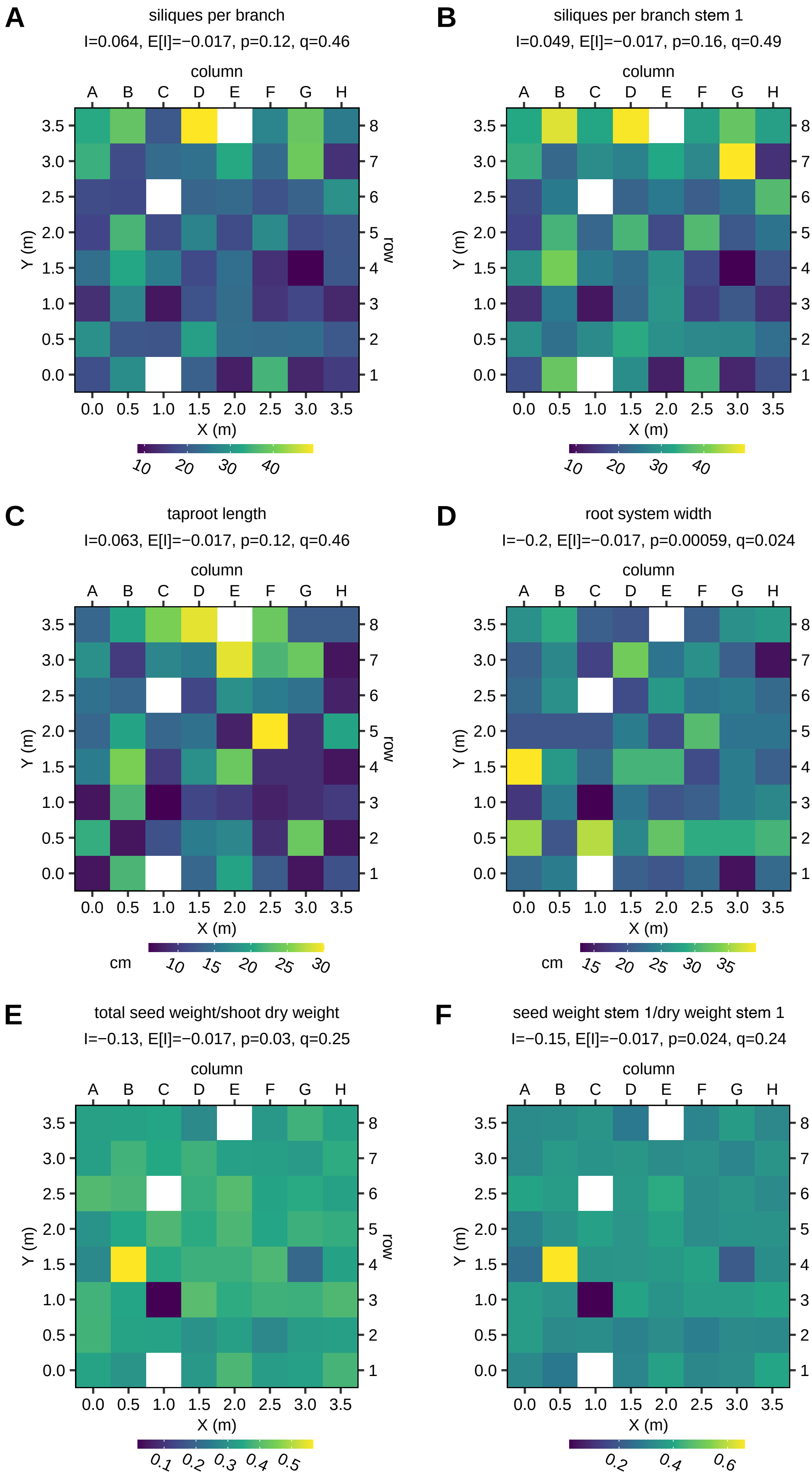
A
siliques per branch
I=0.064, E[I]=−0.017, p=0.12, q=0.46
B
siliques per branch stem 1
I=0.049, E[I]=−0.017, p=0.16, q=0.49
C
taproot length
I=0.063, E[I]=−0.017, p=0.12, q=0.46
D
root system width
I=−0.2, E[I]=−0.017, p=0.00059, q=0.024
E
total seed weight/shoot dry weight
I=−0.13, E[I]=−0.017, p=0.03, q=0.25
F
seed weight stem 1/dry weight stem 1
I=−0.15, E[I]=−0.017, p=0.024, q=0.24
column
A B C D E F G H
row
1 2 3 4 5 6 7 8
Y (m)
X (m)
0.0 0.5 1.0 1.5 2.0 2.5 3.0 3.5
cm

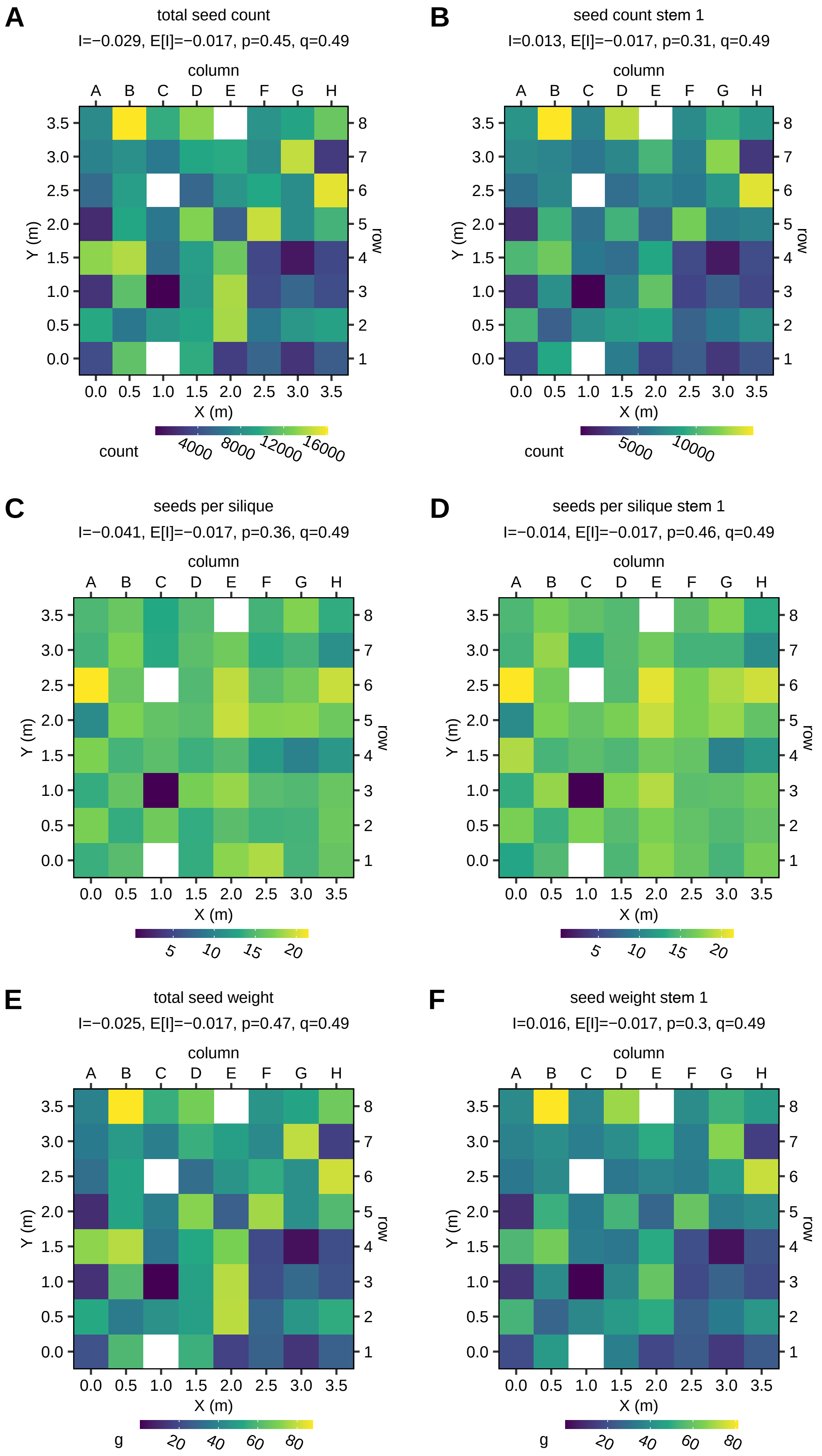
A
total seed count
I=−0.029, E[I]=−0.017, p=0.45, q=0.49
column
A B C D E F G H
row
Y (m)
X (m)
count
4000 8000 12000 16000
B
seed count stem 1
I=0.013, E[I]=−0.017, p=0.31, q=0.49
column
A B C D E F G H
row
Y (m)
X (m)
count
5000 10000
C
seeds per silique
I=−0.041, E[I]=−0.017, p=0.36, q=0.49
column
A B C D E F G H
row
Y (m)
X (m)
5 10 15 20
D
seeds per silique stem 1
I=−0.014, E[I]=−0.017, p=0.46, q=0.49
column
A B C D E F G H
row
Y (m)
X (m)
5 10 15 20
E
total seed weight
I=−0.025, E[I]=−0.017, p=0.47, q=0.49
column
A B C D E F G H
row
Y (m)
X (m)
g
20 40 60 80
F
seed weight stem 1
I=0.016, E[I]=−0.017, p=0.3, q=0.49
column
A B C D E F G H
row
Y (m)
X (m)
g
20 40 60 80

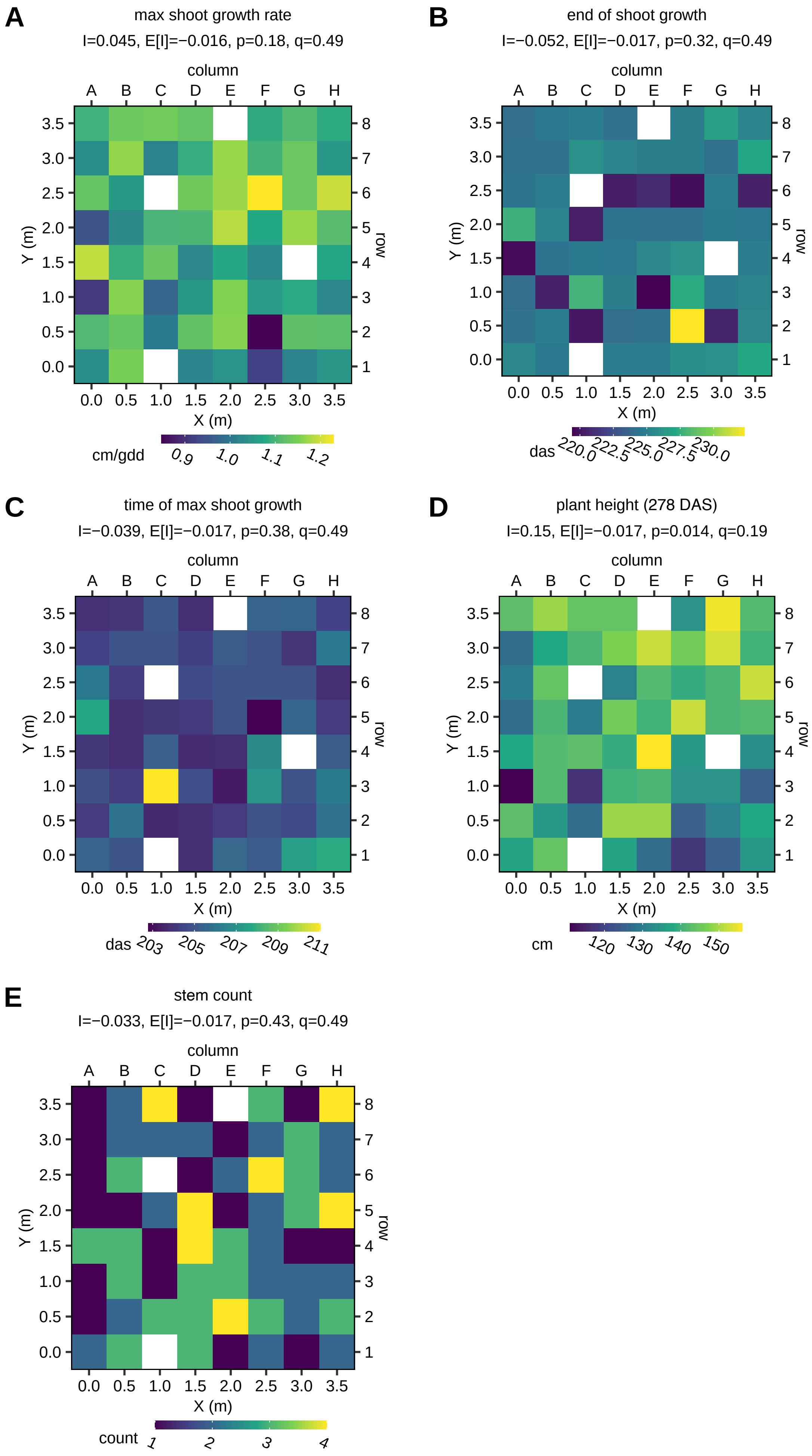
A
max shoot growth rate
I=0.045, E[I]=−0.016, p=0.18, q=0.49
column
A B C D E F G H
Y (m)
X (m)
row
cm/gdd 0.9 1.0 1.1 1.2
B
end of shoot growth
I=−0.052, E[I]=−0.017, p=0.32, q=0.49
das 220.0 222.5 225.0 227.5 230.0
C
time of max shoot growth
I=−0.039, E[I]=−0.017, p=0.38, q=0.49
das 203 205 207 209 211
D
plant height (278 DAS)
I=0.15, E[I]=−0.017, p=0.014, q=0.19
cm 120 130 140 150
E
stem count
I=−0.033, E[I]=−0.017, p=0.43, q=0.49
count 1 2 3 4
