# Supplementary material for: Predicting yield of individual field-grown rapeseed plants from rosette-stage leaf gene expression
Source: PLoS Comput Biol. 2023 May 30;19(5):e1011161. doi: 10.1371/journal.pcbi.1011161 (PMC10256231; doi:10.1371/journal.pcbi.1011161)
Supplement: S4 Fig — (PDF) [file pcbi.1011161.s004.pdf]

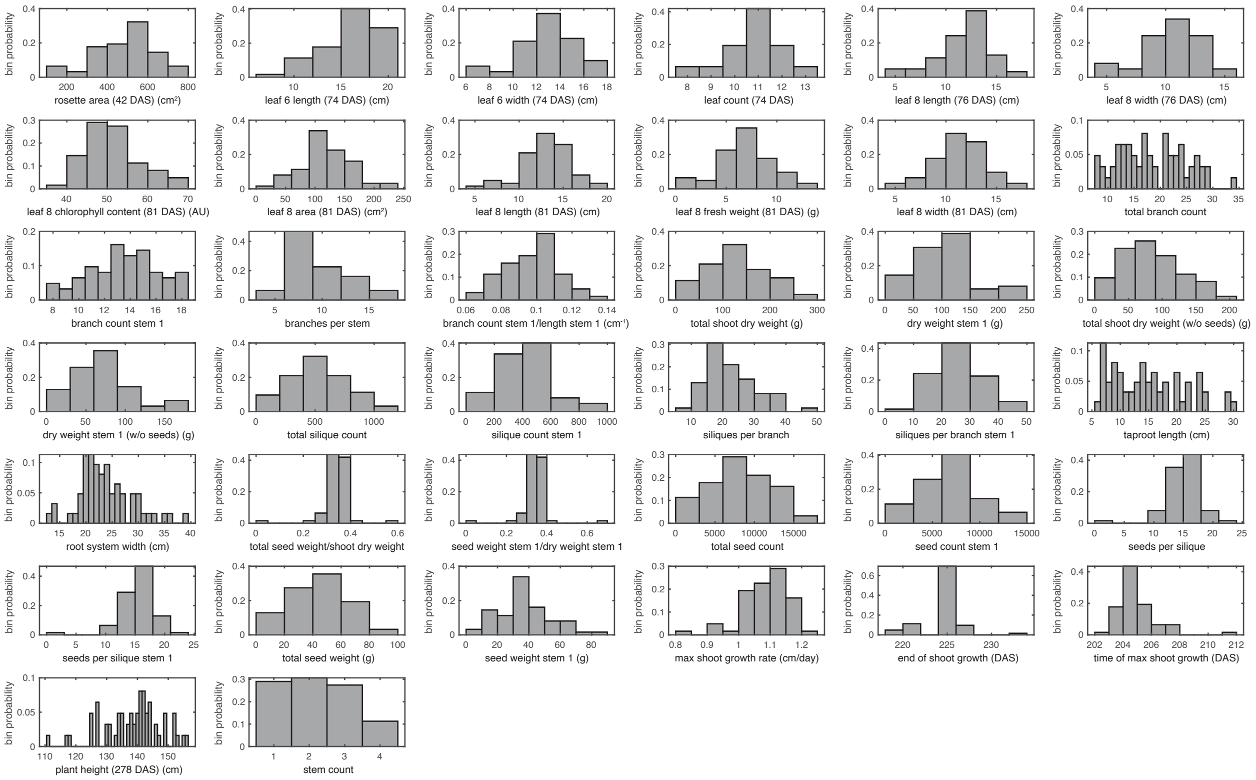

S4 Fig. Phenotype histograms. Histograms were plotted using the 'histogram' function in Matlab R2018b, with automatic binning.
