# Supplementary material for: Predicting yield of individual field-grown rapeseed plants from rosette-stage leaf gene expression
Source: PLoS Comput Biol. 2023 May 30;19(5):e1011161. doi: 10.1371/journal.pcbi.1011161 (PMC10256231; doi:10.1371/journal.pcbi.1011161)
Supplement: S5 Fig — (PDF) [file pcbi.1011161.s005.pdf]

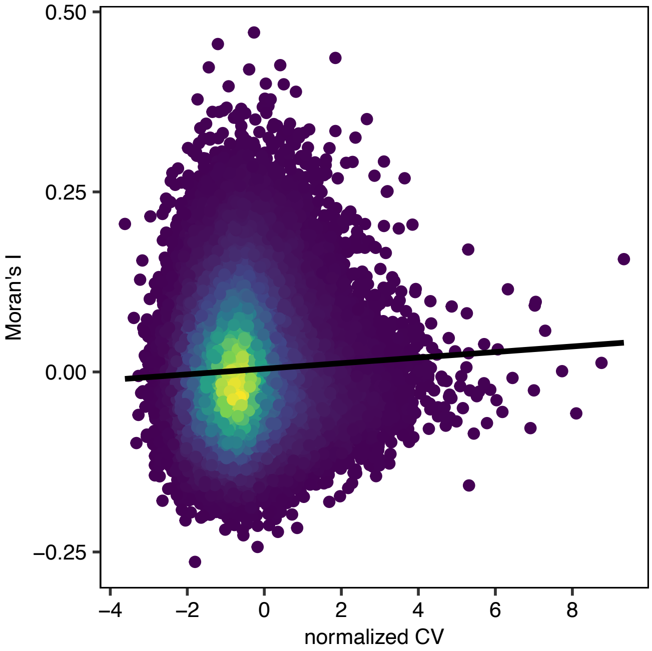

S5 Fig. Heatmap of Moran's I versus *normCV* values of gene expression profiles. Dots represent gene expression profiles and dot colors reflect dot density (yellow = high density, dark blue = low density). The line is an ordinary least-squares linear regression fit.
