# Supplementary material for: Predicting yield of individual field-grown rapeseed plants from rosette-stage leaf gene expression
Source: PLoS Comput Biol. 2023 May 30;19(5):e1011161. doi: 10.1371/journal.pcbi.1011161 (PMC10256231; doi:10.1371/journal.pcbi.1011161)

S6 Fig. Phenotype predictions versus observations. Each plot shows the predicted versus measured values for the best-performing 'all genes' model for a given phenotype (**Table 2**). Qualitative and low-count phenotypes and phenotypes with median test R^2 values < 0 are not shown. Vertical grey lines range from the minimum to the maximum predicted value for a given plant across all model repeats, and colored dots represent predictions for the repeat with the median pooled R^2 score. Different marker colors indicate the 10 different test sets in this repeat. Perfect predictions are located on the dashed diagonal line in each panel.

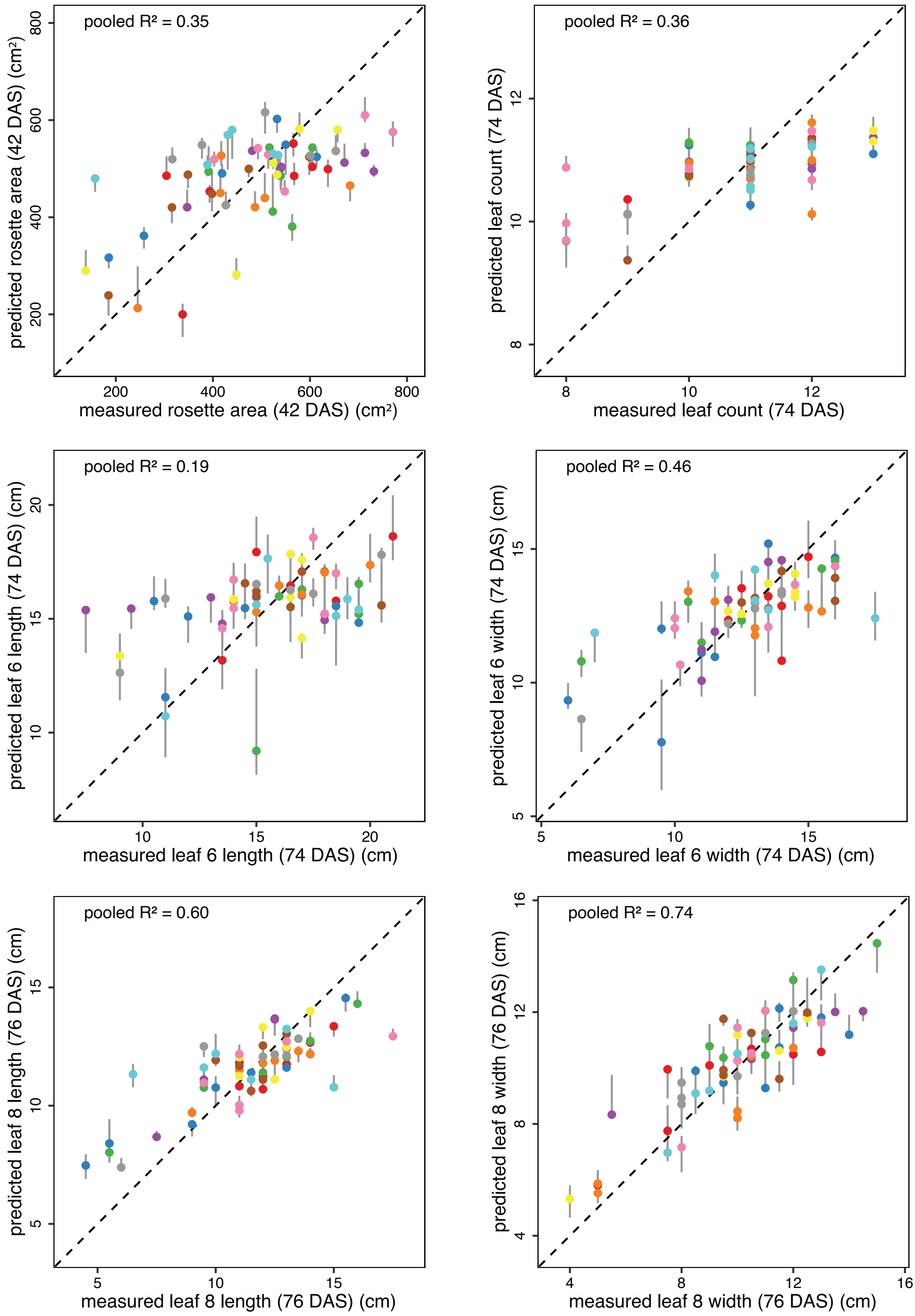

S6 Fig (continued).

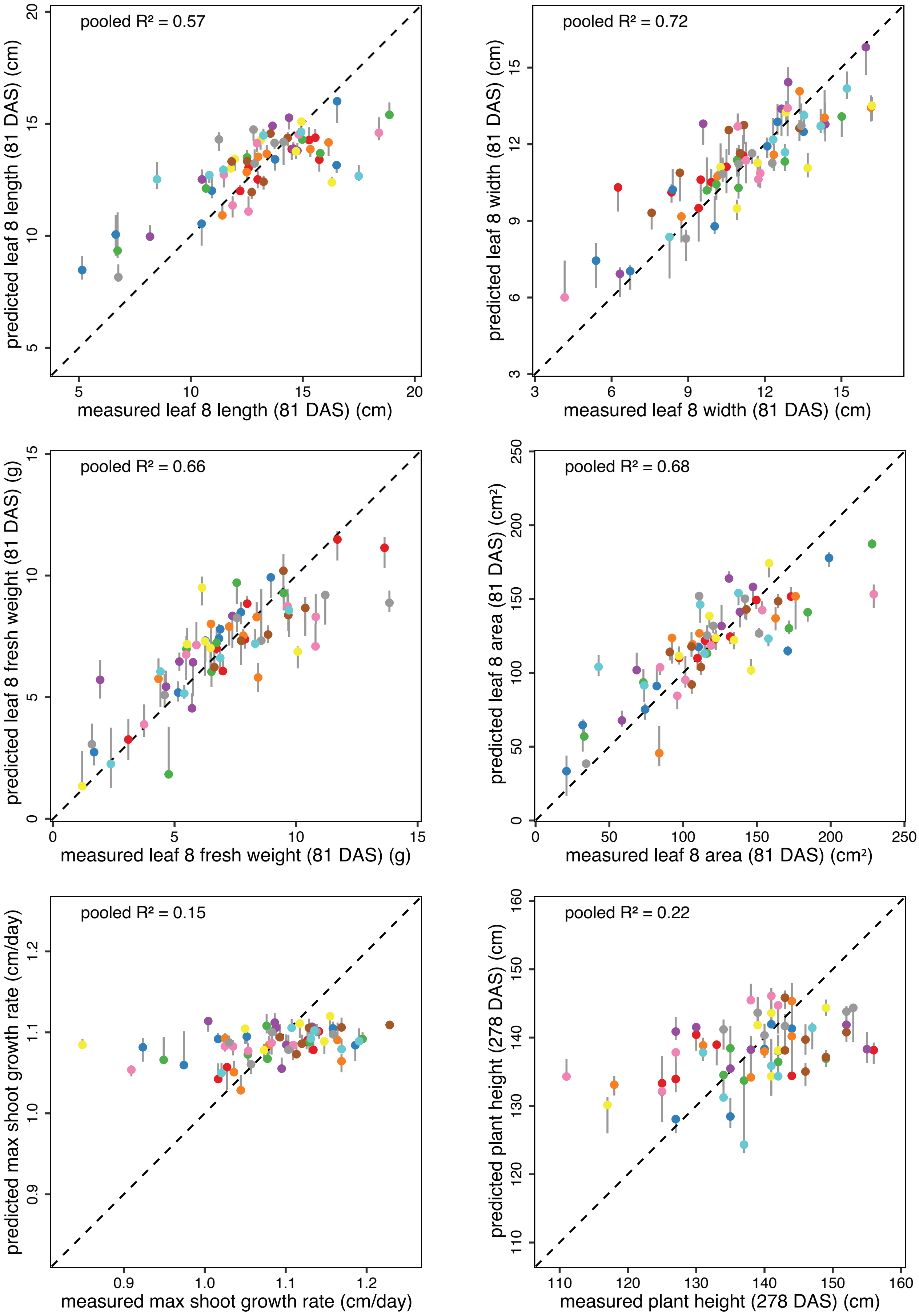

S6 Fig (continued).

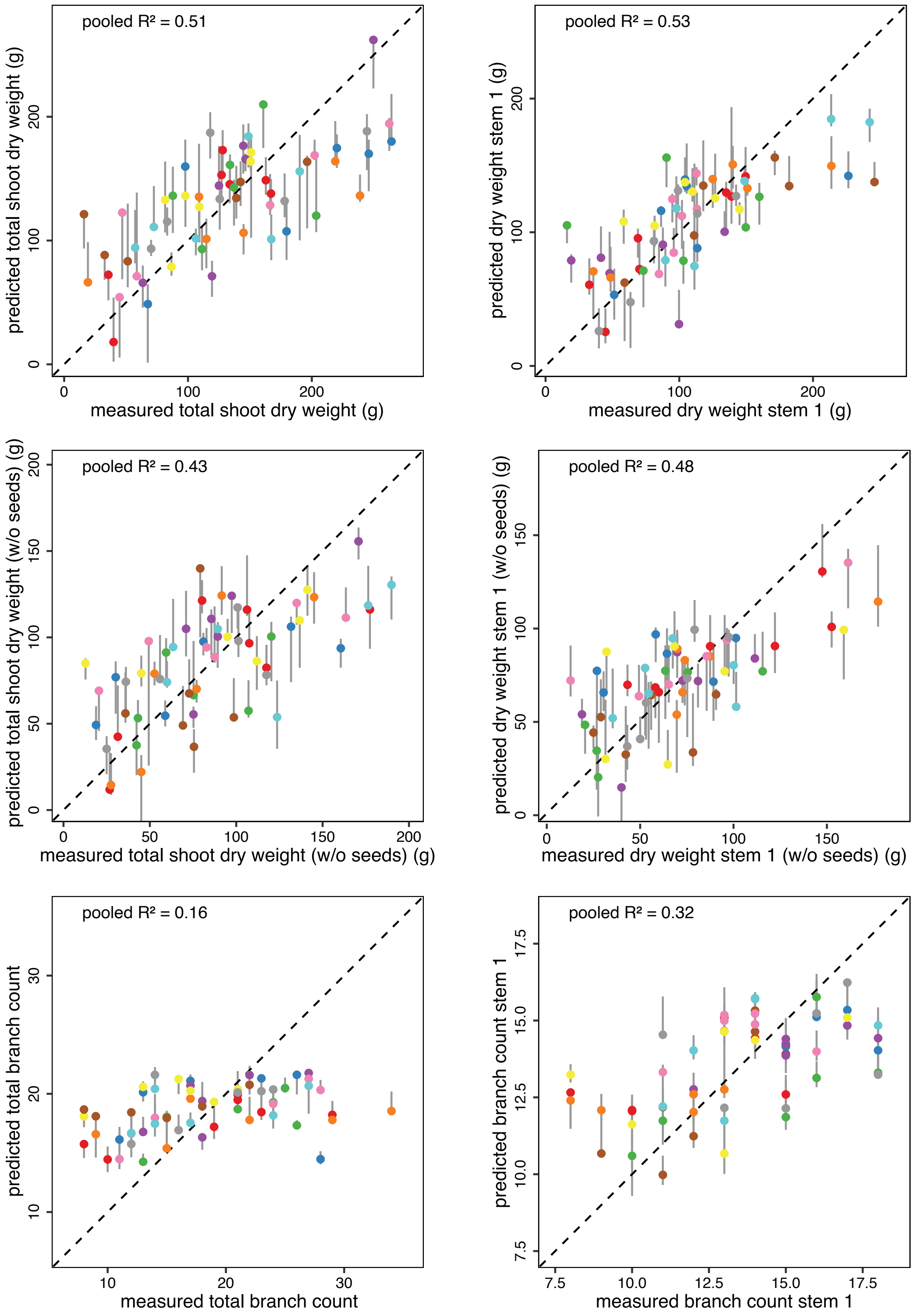

S6 Fig (continued).

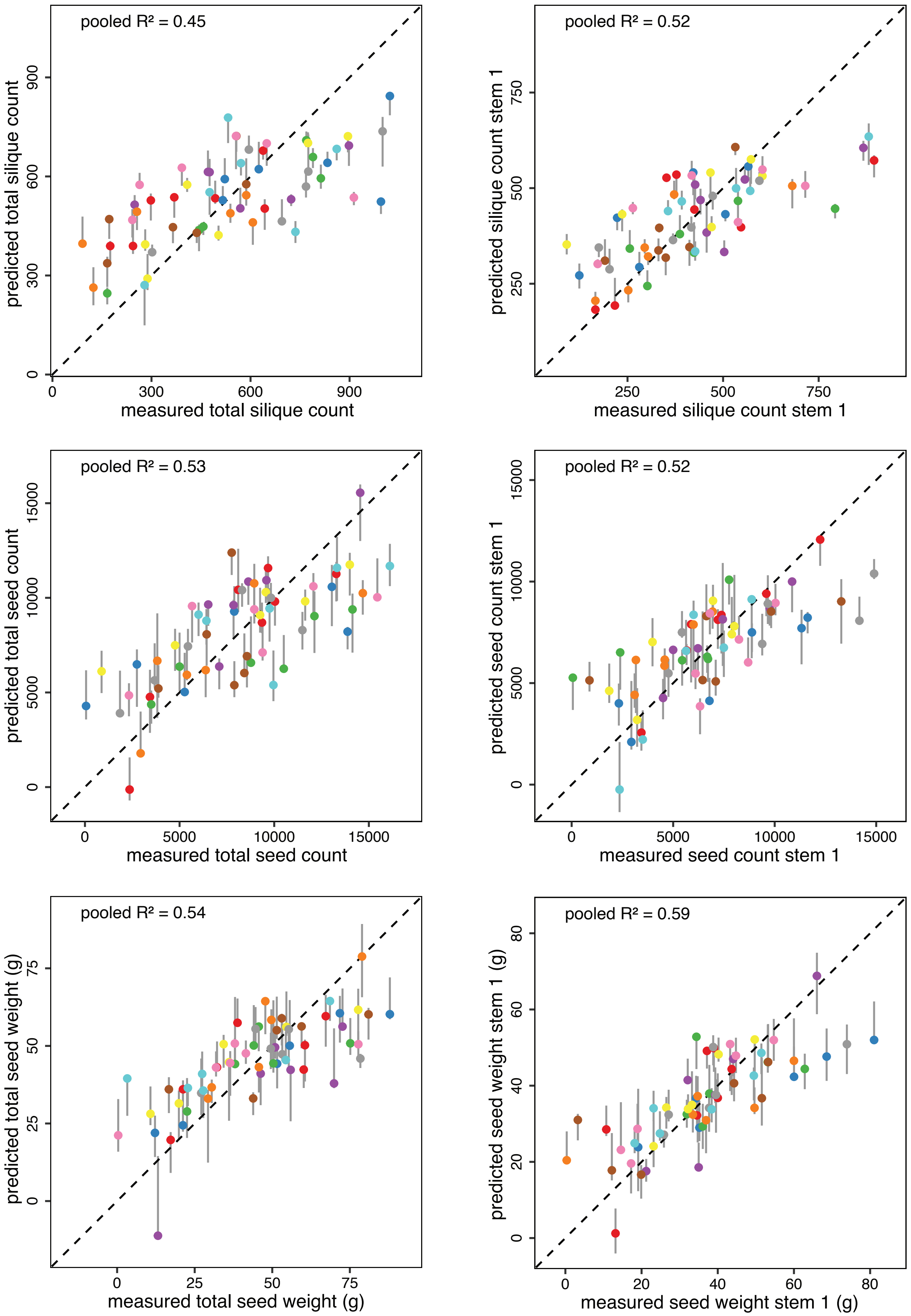

S6 Fig (continued).

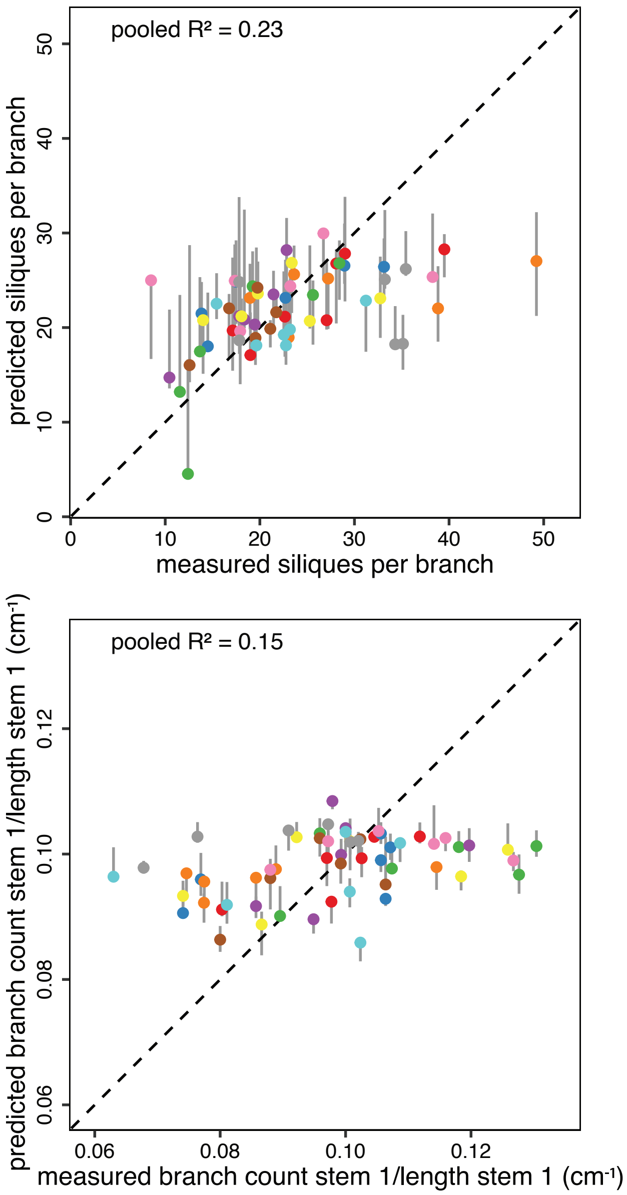

Supplement: S6 Fig — (PDF) [file pcbi.1011161.s006.pdf]
