# Supplementary material for: Predicting yield of individual field-grown rapeseed plants from rosette-stage leaf gene expression
Source: PLoS Comput Biol. 2023 May 30;19(5):e1011161. doi: 10.1371/journal.pcbi.1011161 (PMC10256231; doi:10.1371/journal.pcbi.1011161)
Supplement: S7 Fig — (PDF) [file pcbi.1011161.s007.pdf]

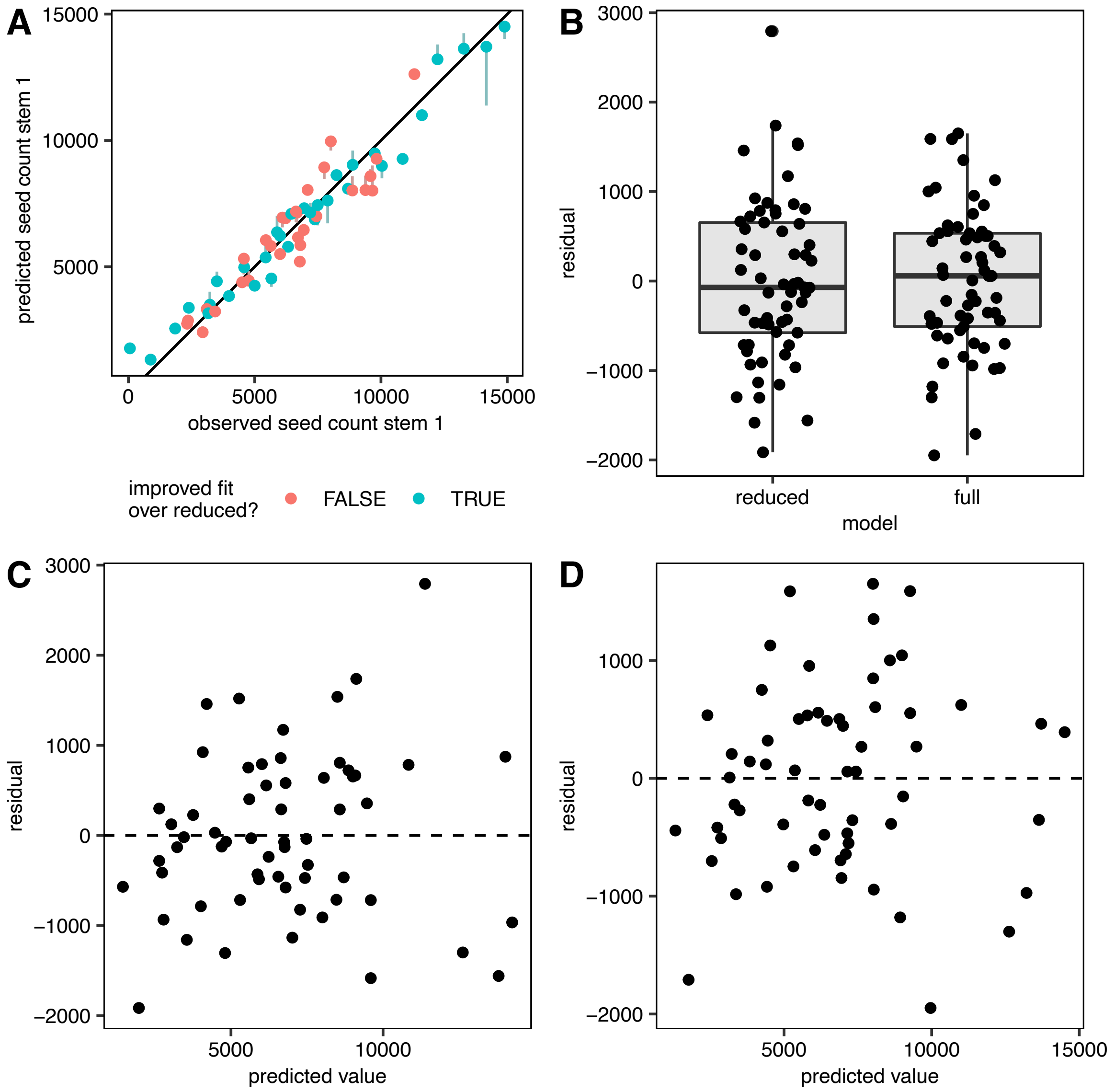


**S7 Fig. Performance of log-link model predicting seed count stem 1 conditioned on silique count stem 1 as a function of expression of the best predictor gene, *BnaCnng56980D*. A.** Plot of predicted versus observed seed counts on stem 1. Values predicted by the full model with heteroscedastic error structure are shown as dots. The ends of the tails attached to the dots indicate the phenotype values predicted by the reduced model (without gene expression effect). Blue dots indicate improved predictions in the full model versus the reduced model, red dots indicate worse predictions in the full model. **B.** Distribution of residuals in the full and reduced models. **C.** Residuals versus predicted values for the reduced model. **D.** Residuals versus predicted values for the full model.
