# Supplementary material for: Predicting yield of individual field-grown rapeseed plants from rosette-stage leaf gene expression
Source: PLoS Comput Biol. 2023 May 30;19(5):e1011161. doi: 10.1371/journal.pcbi.1011161 (PMC10256231; doi:10.1371/journal.pcbi.1011161)
Supplement: S9 Fig — (PDF) [file pcbi.1011161.s009.pdf]

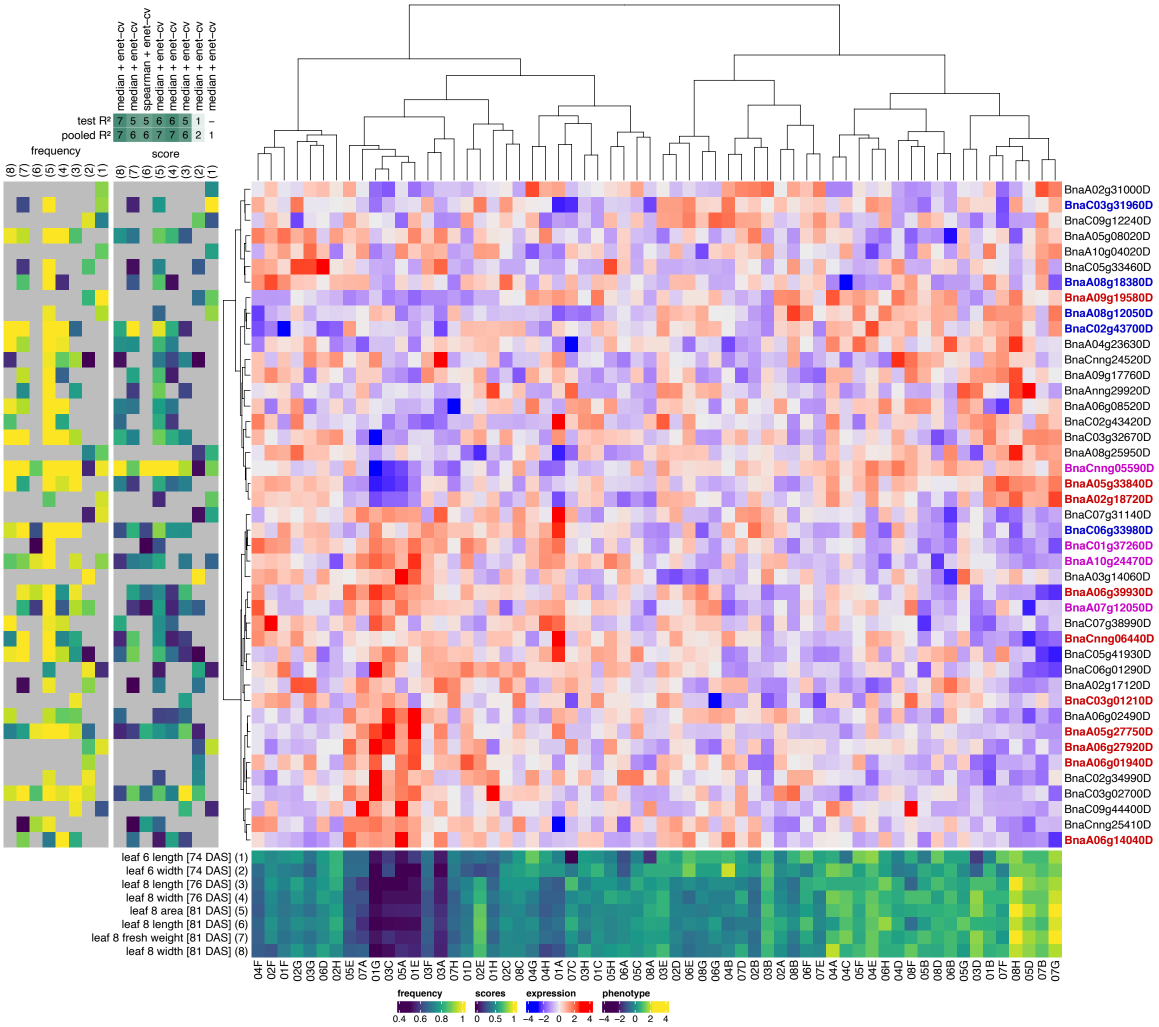

S9 Fig. Top predictor genes in enet models of leaf phenotypes. A clustered heatmap of the z-scored gene expression profiles of the top genes for predicting leaf phenotypes is shown centrally (blue-red color scale, Ward.D2 hierarchical clustering). The leaf phenotypes concerned and their z-scored profiles across plants are shown at the bottom (dark blue-yellow heatmap with plant identifiers at the bottom). For each of these phenotypes, the top-10 most important genes (highest median elastic net coefficients across all 90 cross-validation splits) of the enet model with the highest median test R^2 score are included on the figure (gene identifiers are shown at right). The green-blue score panel to the left of the expression heatmap shows the median elastic net coefficients of the selected genes in each of the selected phenotype models, normalized to the maximum coefficient per model to make the color scales of the different models (columns) comparable. The mostly yellow frequency panel to the left of the score panel shows the frequencies at which genes were selected as features across all 90 cross-validation splits of a given model. Grey squares in the score and frequency panels indicate that a given gene was not selected as a feature in a given model. The phenotypes in the score and frequency panels are identified by numbers (1-8) on top of the panels, corresponding to the numbers associated with the phenotypes in the bottom phenotype panel. On top of the score panel, the feature selection techniques used in the best-scoring enet models for each phenotype are shown (median = selection of features with median rlog gene expression > 0, spearman = Spearman correlation, hsic-5000 = HSIC lasso, see Methods), as well as the corresponding test and pooled R^2 scores rounded to the nearest 0.1 and then multiplied by ten (e.g. a test R^2 score of 0.38 would be denoted as 4). Genes that are also found in the top-10 RF predictor lists for leaf phenotypes (**Fig 3**) are highlighted in red, while genes that are also found in the top-10 enet or RF predictor lists for yield phenotypes (**Figs 4** and **S10**) are highlighted in blue. Genes found in both the top-10 RF predictor lists for leaf phenotypes and the top-10 enet or RF predictor lists for yield phenotypes are highlighted in magenta.
