# Supplementary material for: Predicting yield of individual field-grown rapeseed plants from rosette-stage leaf gene expression
Source: PLoS Comput Biol. 2023 May 30;19(5):e1011161. doi: 10.1371/journal.pcbi.1011161 (PMC10256231; doi:10.1371/journal.pcbi.1011161)
Supplement: S10 Fig — (PDF) [file pcbi.1011161.s010.pdf]

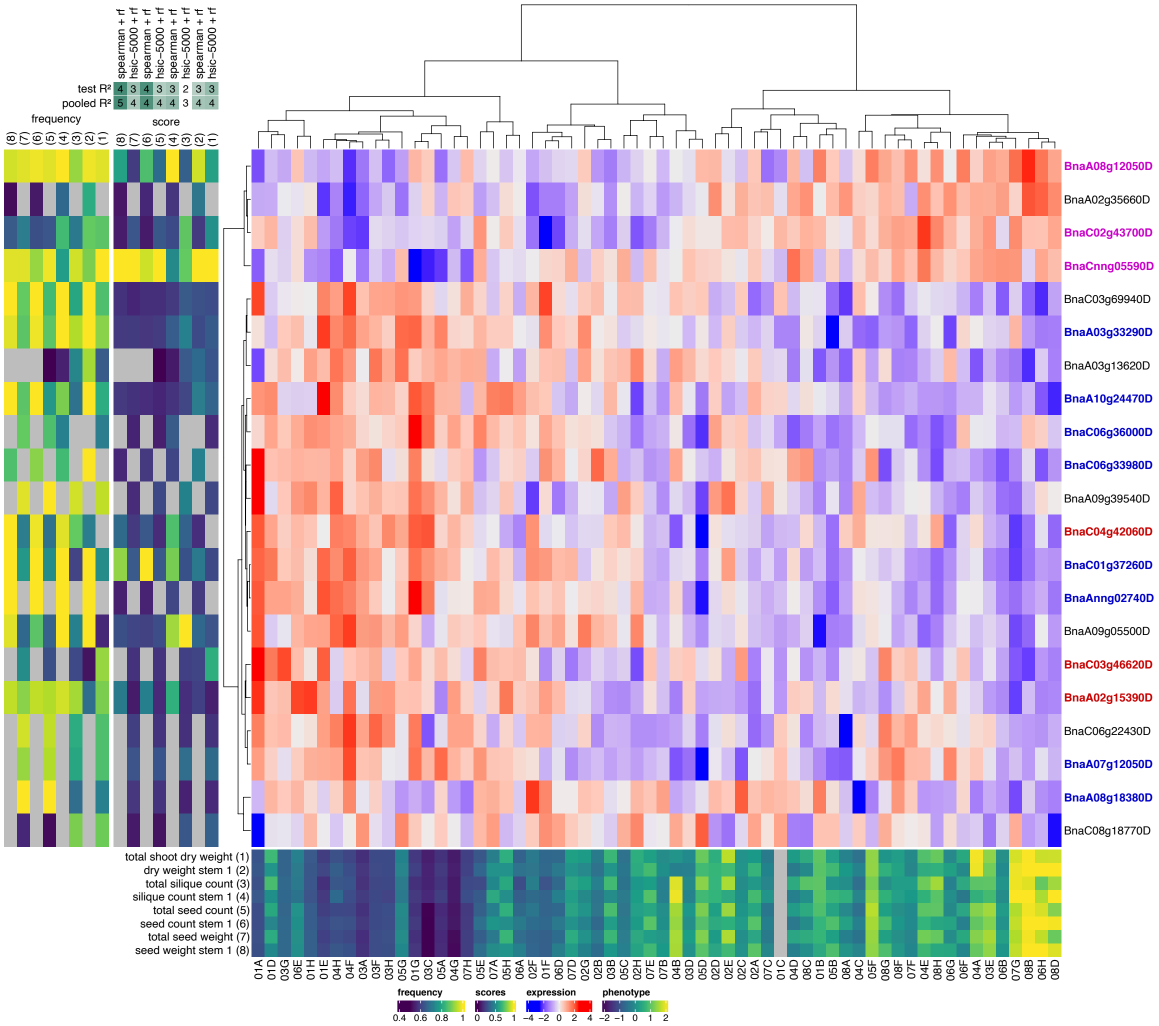

S10 Fig. Top predictor genes in RF models of yield phenotypes. A clustered heatmap of the z-scored gene expression profiles of the top genes for predicting yield phenotypes is shown centrally (blue-red color scale, Ward.D2 hierarchical clustering). The yield phenotypes concerned and their z-scored profiles across plants are shown at the bottom (dark blue-yellow heatmap with plant identifiers at the bottom). For each of these phenotypes, the top-10 most important genes (highest median gini importance across all 90 cross-validation splits) of the RF model with the highest median test R^2 score are included on the figure (gene identifiers are shown at right). The mostly blue score panel to the left of the expression heatmap shows the median gini importance scores of the selected genes in each of the selected phenotype models, normalized to the maximum importance score per model to make the color scales of the different models (columns) comparable. The yellow-green frequency panel to the left of the score panel shows the frequencies at which genes were selected as features across all 90 cross-validation splits of a given model. Grey squares in the score and frequency panels indicate that a given gene was not selected as a feature in a given model. The phenotypes in the score and frequency panels are identified by numbers (1-8) on top of the panels, corresponding to the numbers associated with the phenotypes in the bottom phenotype panel. On top of the score panel, the feature selection techniques used in the best-scoring RF models for each phenotype are shown (median = selection of features with median rlog gene expression > 0, spearman = Spearman correlation, hsic-5000 = HSIC lasso, see Methods), as well as the corresponding test and pooled R^2 scores rounded to the nearest 0.1 and then multiplied by ten (e.g. a test R^2 score of 0.38 would be denoted as 4). Genes that are also found in the top-10 enet predictor lists for yield phenotypes (**Fig 4**) are highlighted in red, while genes that are also found in the top-10 enet or RF predictor lists for leaf phenotypes (**Figs 3** and **S9**) are highlighted in blue. Genes found in both the top-10 enet predictor lists for yield phenotypes and the top-10 enet or RF predictor lists for leaf phenotypes are highlighted in magenta.
