# Supplementary material for: Predicting yield of individual field-grown rapeseed plants from rosette-stage leaf gene expression
Source: PLoS Comput Biol. 2023 May 30;19(5):e1011161. doi: 10.1371/journal.pcbi.1011161 (PMC10256231; doi:10.1371/journal.pcbi.1011161)
Supplement: S11 Fig — (PDF) [file pcbi.1011161.s011.pdf]

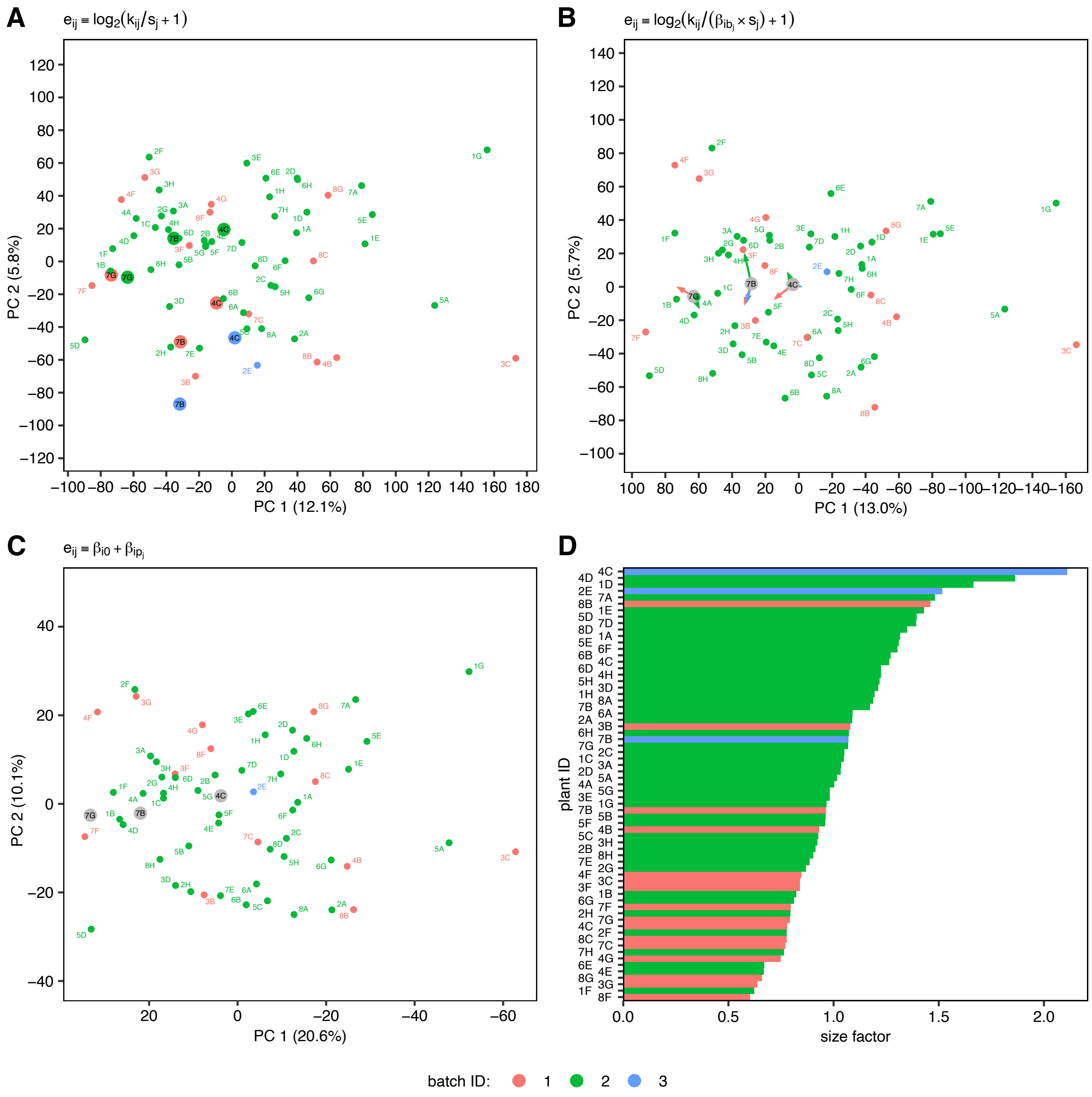

S11 Fig. Sequencing batch effects on RNA-seq count data. A-C. Effect of RNA-seq batch on gene expression in principal component (PC) space. Samples are colored by batch ID and labeled with plant IDs. Samples included in several batches are indicated with bigger dots and black labels. **A.** First two PCs of $\log_2$-tranformed library size-corrected expression profiles, before batch correction. Repeats of samples 4C and 7B are spaced far apart in PC 2, but align well on PC 1. **B.** First two PCs of $\log_2$-transformed library size-corrected and batch-corrected expression profiles. Grey dots indicate corrected expression profiles that were averaged across repeats before log-transforming, and colored arrows point to the batch-specific positions of the samples concerned before averaging. Comparison with **A** shows that batch correction diminishes but does not completely eliminate the gene expression differences between sample repeats. The non-log-transformed version of the data in **B** was used for variance analysis (see Methods). **C.** First two PCs of gene expression profiles obtained through the modified *rlog* transformation, which accounts for library size and batch effects and unites technical repeats in one estimate (grey dots, see Methods). **D.** Size factors estimated by DESeq2 for each sample and batch.
