# Supplementary material for: Predicting yield of individual field-grown rapeseed plants from rosette-stage leaf gene expression
Source: PLoS Comput Biol. 2023 May 30;19(5):e1011161. doi: 10.1371/journal.pcbi.1011161 (PMC10256231; doi:10.1371/journal.pcbi.1011161)
Supplement: S12 Fig — (PDF) [file pcbi.1011161.s012.pdf]

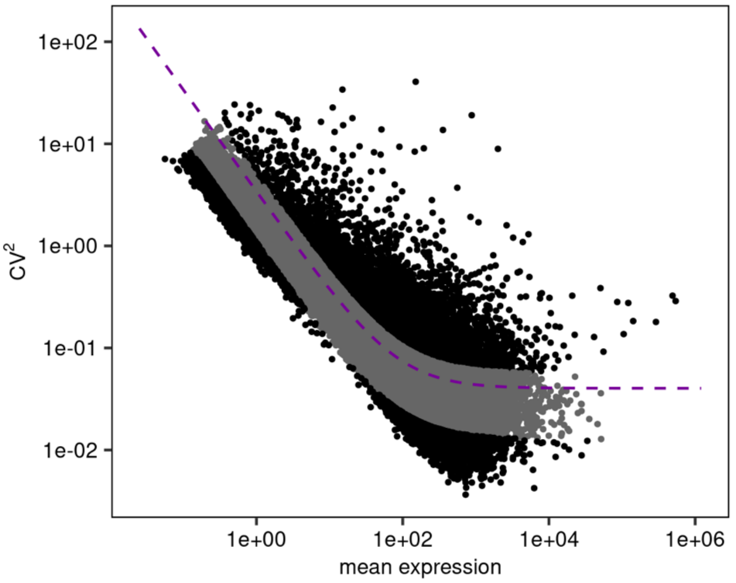

S12 Fig. Gene expression variability in the *B. napus* single-plant dataset. The squared CV is plotted versus the mean expression for genes expressed in ≥ 10 samples (dots). A fitted trendline (see Methods) is shown in purple, the top and bottom 10% of genes ranked by normalized CV (*normCV*, see Methods) are shown in black.
