# Supplementary material for: Predicting yield of individual field-grown rapeseed plants from rosette-stage leaf gene expression
Source: PLoS Comput Biol. 2023 May 30;19(5):e1011161. doi: 10.1371/journal.pcbi.1011161 (PMC10256231; doi:10.1371/journal.pcbi.1011161)
Supplement: Attachment — Submitted filename: reponse_to_reviewers.docx [file pcbi.1011161.s022.docx]

Dear Reviewers, dear Editor,

We thank the reviewers for their appreciative and useful comments. We addressed all comments to the best of our abilities in the revised version of the manuscript. Below, you find our detailed point-by-point responses to all comments in blue font, in between the reviewer’s comments in black font (numbering is ours). We hope these responses satisfactorily address the points raised by the reviewers.

Yours sincerely,

Steven Maere

**Reviewer #1:**

In this interesting paper, the authors use single plant-omics to predict yield of individual field-grown rapeseed plants from rosette-stage 2 leaf gene expression. This is interesting as it suggests a way of predicting crop yield from the expression state of younger plants. They use machine learning to successfully predict the phenotypes of individual B. napus plants from rosette-stage leaf gene expression data. The authors have generated a huge amount of data on individual plants and have carefully compared early stage gene expression and phenotype with final yield. It is nice that the top predictive genes from their models are often linked to the juvenile-adult phase change in Arabidopsis. I think the topic and material will be of interest to PloS computational Biology readers, and the data will be useful to researchers who want to dig into the topic. The authors should be commended for making so much of their data and code available online. I have the following points:

Major point.

**Comment 1.1:** My one major problem with the paper is I found it difficult to understand from the main text how the machine learning approach was applied, how the techniques were chosen, and to get an intuitive understanding of the results and how they can be validated. This wasn’t completely fixed by going to the methods, which explained in detail the approach, but not how the approach was chosen in the first place. This can be simply fixed with a few more explanations.

For example, the authors write: ‘We built random forest (RF) and elastic net (enet) models to predict the phenotypes of individual plants from their autumnal leaf 8 transcriptome, using either all genes or only transcription factors (TFs) as potential features and using three different feature selection techniques (see Methods). For each combination of phenotype, model type (RF or enet), potential feature set (all genes or TFs) and feature selection technique, 9 repeat models were learned, each time using 10-fold cross-validation with different splits (see Methods), resulting per combination in a total of 90 test sets and 9 test set predictions per plant.

It should be explained in the main text the differences between the single gene and multi-gene models, and why random forest and elastic net models are used (why both?). It would also be helpful to explain how the multi-gene models are implemented and what assumptions were used.

**Reply 1.1:** We apologize for any unclarities in our modeling strategy. We now added clarifications in the results section on the motivation for using both single-gene models and multi-gene models and the differences between both, as well as on the reason why we used both random forest and elastic net models in the multi-gene scenario. More details on how the multi-gene models are implemented can be found in the Methods section.

In summary, linear mixed-effects models modeling a given phenotype as a function of the expression of a single gene (single-gene models) were used to assess the relation between each gene expression profile and each phenotype separately, in order to get an overall view on which genes’ expression significantly relates to which phenotypes. Linear mixed-effects models were used for this instead of ordinary linear models because they can take into account spatial autocorrelation effects, which can bias significance testing in ordinary linear models. Linear mixed-effects models offer a robust statistical framework for testing the significance of gene expression ‘effects’ (not meant in a causal sense here) on phenotypes in the presence of spatial autocorrelation, also for small sample sizes (in our case 62 plants).

Because a single gene expression profile may not be sufficient to fully ‘explain’ (not causal) a phenotype profile, we additionally used multi-gene models, i.e. models using multiple gene expression profiles as explanatory features, to model phenotypes. As the potential feature space (76,808 genes) is huge compared to the number of data points (62 plant samples), simply using ordinary linear models or linear mixed-effects models with the expression of all genes as features makes no sense, as there’s a myriad different ways to perfectly explain a given phenotype for 62 plants from 76,808 gene expression profiles. Most of these models will be seriously overfit and will generalize badly to unseen data. Instead, we use elastic net and random forest models. Elastic net (enet) is a regularized regression method that uses a penalty on the sum of feature coefficient magnitudes (L1-norm, as in lasso) and an additional penalty on the magnitude of the coefficient vector (L2-norm or Euclidean norm, as in ridge regression) to shrink the feature coefficients and limit the amount of features used to explain an outcome variable, with the purpose of enhancing the generalizability of the resulting model to unseen data. Random forest (RF) reduces overfitting by training a ‘forest’ of decision trees, in which each tree is trained on a dataset sampled with replacement from the original data, and at each tree split only a random subset of features are taken into account as possible predictors. The predicted outcome for a given input is then taken to be the average of the output of the different trees in the forest.

Models were learned using either the set of all genes (n=76,808) or the (much smaller) set of all transcription factors (TFs, n=2,521) as features. To further limit the number of features used to train the machine learning models, we use feature selection to select a priori some subset of features that are potentially most interesting, e.g. gene expression features that are significantly correlated with the phenotype of interest. As different feature selection techniques may have different biases and we don’t know a priori which one is best for the problem at hand, we used three different feature selection techniques.

The main reason for using both elastic net and random forest models is that the former can only capture linear relationships in the data, while the latter can also capture non-linear effects. More generally, different modeling techniques have different biases and it is hence always a good idea to use several and see which one works best and to what extent they agree.

The enet and RF models are used in a 10-fold cross-validation setup (i.e. the data was split the in 10 data subsets, each set was selected in turn as the test set, the model was trained on the remaining 9 sets and applied to the test set) to assess to what extent the models generalize to unseen (test set) data. To assess the variance of the test set predictions, the 10-fold cross-validation procedure is repeated 9 times, giving rise to 90 test sets and 9 test set predictions per plant for each combination of phenotype, model type (RF or enet), potential feature set (all genes or TFs) and feature selection technique. The best model for a given phenotype and potential feature set was taken to be the one with the highest median test *R*^2^ value across all 90 test sets for continuous and high-count phenotypes, or the highest median test accuracy for qualitative or low-count discrete phenotypes.

Minor points

**Comment 1.2:** The authors write in lines 193-206, ‘ In a previous study on a similar number of field-grown maize plants [28], 14.17% of transcripts were found to be significantly spatially autocorrelated at q ≤ 0.01, which is considerably more than the 0.22% recovered here at q ≤ 0.05. This may be due to differences in the way Moran’s I values and their significance were calculated in Cruz, De Meyer (28) versus the present study (see Methods).
Reference 28 appears to be the from the same group as this paper. Wouldn’t it be possible to get the data from 28 and do the same test? The methods dont seem to explain the differences in how the Moran's I values were calculated between the two papers I don’t fully understand what was done after this in lines up to 206, and what the evidence is that there is spatial patterning in the new data? I don’t think it matters for this papers results whether there is spatial patterning, but I found this part of the paper confusing.

**Reply 1.2:** The reviewer makes a valid point that we should have investigated the observed differences in the numbers of significant Moran’s I values between our previous maize study and the present study in more detail. We now applied both the method from the maize study (inverse distance-based spatial weight matrix, parametric testing using the R package Ape v:5.7) and the method we use in the present study (queen contiguity-based spatial weight matrix, permutation-based testing) on both datasets (see below).

While doing so, we found that we previously made a mistake in defining the queen contiguity-based spatial weight matrix, by setting the weight of each plant to itself to 1 instead of 0. This affects the calculated Moran’s I values, but as there is a perfect linear correlation between the incorrect and correct Moran's I values, the ordering of genes according to Moran's I is not affected, and therefore the follow-up analysis (Mann-Whitney U based GO analysis) and conclusions are also not affected.

Given that permutation tests were used to assess the significance of Moran’s I values, in theory none of the reported *p*-values for gene expression Moran’s I are affected by our mistake, since the mistake has the same linear transformation effect on both the Moran’s I values calculated on the real data and the Moran’s I values calculated on permuted data. We have verified this empirically by rerunning the permutation tests, and found that besides small deviations due to the random nature of the permutations used, there was no meaningful difference in the computed *p*-values. However, due to these small deviations, only 140 instead of originally 169 genes were found to have significantly spatially autocorrelated expression profiles at *q* ≤ 0.05.

The aforementioned perfect linear correlation only holds when all variables are measured on the same points on the field, which is the case for the gene expression values. Some phenotypes have missing data points however. As a result, there are small differences in the Moran’s I ranking of the phenotypes with respect to the previous ranking (leaf count (74 DAS) moves up 1 rank and leaf 6 lesions (74 DAS) too, see **S2 Data** (formerly **Data S2**) sheet D).

All tables and figures that were affected by this mistake have been corrected: **S2 Data** (formerly **Data S2**) sheets A and D, **S3 Fig** (formerly **Figure S2**) and **S5 Fig** (formerly **Figure S4).**

Below is a summary of the results obtained with the method used for the maize dataset of Cruz, De Meyer et al (2020) (‘asInMaize’) and the (corrected) method used in the present study (‘asInBrassica’) on both the maize and *B. napus* gene expression datasets. Please note that application of the ‘asInBrassica’ method with queen contiguity-based spatial weight matrix to the Cruz, De Meyer et al (2020) data is complicated by the fact that it is more difficult to define 'neighboring plants' in the maize dataset. This is because, as opposed to the present *B. napus* trial, there was no equispaced grid structure in the maize field trial, which instead used rows of maize plants with 75 cm inter-row spacing and 10 cm inter-plant distance within rows. We therefore defined all plants within a distance of 91 cm of each other as neighbors, 91 cm being the minimal distance possible without having maize plants with zero neighbors (only 60 selected plants were expression profiled in the maize field trial).

Before multiple testing correction, the two methods yield about the same relative amount (%) of significantly autocorrelated gene expression profiles at *p* ≤ 0.05, both for the maize dataset and for the *B. napus* dataset, and there’s only about a two-fold difference in the percentage of genes identified as spatially autocorrelated in maize versus *B. napus*. For both maize and *B. napus*, Benjamini-Hochberg multiple testing correction has a stronger effect on the percentage of genes identified as significantly spatially autocorrelated using the ‘asInBrassica’ method than on the percentage identified using the ‘asInMaize’ method, indicating that the sets of *p*-values identified by both methods are structurally different. Multiple testing correction effects are also more severe for *B. napus*, as more tests are performed for this species (76,808 in *B. napus* versus 18,171 in maize). We therefore conclude that the difference between the percentages of significantly spatially autocorrelated gene expression profiles observed in the maize study and the present study is mainly due to methodological differences and a stronger effect of multiple testing correction on the *B. napus* dataset.

Maize dataset:

`asInMaize`: 2,574 / 18,171 genes significantly autocorrelated (14.17%) at *q* ≤ 0.01

`asInMaize`: 3,993 / 18,171 genes significantly autocorrelated (21.97%) at *q* ≤ 0.05

`asInMaize`: 5,764 / 18,171 genes significantly autocorrelated (31.72%) at *p* ≤ 0.05

`asInBrassica`: 716 / 18,171 genes significantly autocorrelated (3.94%) at *q* ≤ 0.01

`asInBrassica`: 2,464 / 18,171 genes significantly autocorrelated (13.56%) at *q* ≤ 0.05

`asInBrassica`: 5,759 / 18,171 genes significantly autocorrelated (31.69%) at *p* ≤ 0.05

Brassica napus dataset:

`asInMaize`: 2,556 / 76,808 genes significantly autocorrelated (3.33%) at *q* ≤ 0.01

`asInMaize`: 5,371 / 76,808 genes significantly autocorrelated (6.99%) at *q* ≤ 0.05

`asInMaize`: 11,586 / 76,808 genes significantly autocorrelated (15.08%) at *p* ≤ 0.05

`asInBrassica`: 0 / 76,808 genes significantly autocorrelated (0.00%) at *q* ≤ 0.01

`asInBrassica`: 140 / 76,808 genes significantly autocorrelated (0.18%) at *q* ≤ 0.05

`asInBrassica`: 11,945 / 76,808 genes significantly autocorrelated (15.55%) at *p* ≤ 0.05

Despite the fact that the ‘asInMaize’ method yields more spatially autocorrelated genes at *q* ≤ 0.05 than the ‘asInBrassica’ method, we prefer to stick with the ‘asInBrassica’ method in the current study, as the parametric testing used in the ‘asInMaize’ method assumes a normal distribution of the Moran's I value under the assumption of no spatial autocorrelation, which we found does not always hold for real data. Therefore we consider the permutation testing used in the ‘asInBrassica’ method (which does not make such assumptions) to be more reliable. As for the weight matrix used, there are no fundamental reasons to prefer one or the other. Both methods generate largely similar Moran’s I rankings (Spearman rank correlation = 0.8761 for gene expression profiles, Spearman rank correlation = 0.8645 for phenotypes).

For completeness, we now added the Moran’s I values and the corresponding *p*- and *q*-values calculated by the ‘asInMaize’ method on the *B. napus* data to **S2 Data** sheets A and D, in addition to the (corrected) ‘asInBrassica’ values. Results using the ‘asInBrassica’ and the original ‘asInMaize’ method on the maize gene expression dataset of Cruz, De Meyer et al. (2020) can be found in **S1 Table for Reviewers.xlsx**. As the latter results have no relevance for the present study, they are not meant to be included in the manuscript or supplementary.

**Comment 1.3:** Lines 935/936 I found it interesting that seedling emergence was a poor predictor of yield ‘ the seedling emergence date was not recorded in the present field trial, but the closest proxy that was measured, namely rosette area at 14 DAS, was found to be a bad predictor for yield, indicating that variation in seed germination and seedling emergence across the field did not by themselves have a major impact on yield.’ I note in the methods that ‘Early- and late-emerging seedlings were pruned preferentially (based on visual assessment) to make the remaining seedling population as homogeneous as possible.’ Should the authors clarify that in this work they cant assess the effects of seedling emergence on yield as they prune early and late emerging seedlings?

**Reply 1.3:** The reviewer is correct that the pruning of early-and late-emerging seedlings at every grid location (among the maximum three seedlings that emerged there) rendered seedling emergence less variable in our trial. There was however undoubtedly still residual variation in seedling emergence. Our results suggest that this residual variation did not have a major impact on yield in the present trial, which is what we aimed to convey in the text. The reviewer however correctly points out that, given the pruning performed, we cannot conclude anything from our trial about more general effects of seedling emergence on *B. napus* yield. We now added the following sentence in the text to make this clearer: ‘It should be noted however that the variation in seedling emergence in the present trial was mitigated by preferential pruning of early- and late-emerging seedlings at every grid position (see Methods), rendering our trial unsuited to assess the effects of seedling emergence on yield in general.’

**Comment 1.4:** Also, is the data showing that rosette area at 14DAS was a bad predictor for yield in the paper? (sorry if I missed it, but if so maybe reference where this data is shown here as well?)

**Reply 1.4:** This is shown in **Fig 5** and **S9 Data** (formerly **Data S8**): using rosette area (14 DAS) as a predictor yields negative median test *R*^2^ values for all yield phenotypes except total branch count (median test *R*^2^=0.07). A negative test *R*^2^ value indicates that the model does worse than a model in which each test sample is predicted to have as phenotype the mean of all test samples. We have now referenced **Fig 5** and **S9 Data** in the discussion section concerned.

**Reviewer #2**:

The study by De Meyer et al., investigates the phenotypes and gene expression in rapeseed plants, with the aim to predict spring phenotypes given autumn data. The authors found that single-plant omics can be used to identify genes and processes influencing crop yield in the field. Overall, the work is interesting, well-executed, and the topic exciting. Overall, we would be happy to see this paper published. However, there are some places that need clarification:

**Comment 2.1:** Figure 1: ‘Principal component analysis (PCA) suggests that there are no subpopulations of plants with distinct expression or phenotype profiles’. It would be good to have statistics support this. For example, is there a correlation between the distance of plants in the field and the points in the PCA plot?

**Reply 2.1:** We merely meant to say here that there are no clearly separated clusters of plants in the PCA plots, which would be indicative of subsets of plants that cannot be considered part of the same population (which could happen as a result of e.g. subsets of plants having a different genetic makeup, or as a result of e.g. different parts of the field having abruptly dissimilar soil conditions so that plants grown in different parts of the field could be considered to undergo separate, discrete treatments). We’ve added the words ‘clearly separated’ before subpopulations in the sentence concerned to make this clearer.

As suggested, we have now analyzed correlations between the distance of plants in the field and their distance in transcriptome and phenotype space, but using the distances in the full transcriptome and phenotype spaces rather than distances on the PCA plots, as the PCA plots are only 2D representations of the real data. There are indeed weak but significant correlations between the distance of plants in the field and their distance in transcriptome and phenotype space (see new **S2 Fig**), indicative of spatial autocorrelation effects. These spatial autocorrelation effects however do not lead to a separation of the plant population in discrete, clearly distinct groups of plants. We thank Dr. Mutwil for the suggestion to incorporate distance correlation statistics as this gives a better indication of spatial autocorrelation effects than visualizing the field coordinates on the PCA plots, as we did previously.

**Comment 2.2:** Line 195: The authors mention that Moran’s I is calculated differently in this study. Why? Please elaborate.

**Reply 2.2:** Please see our reply to **Comment 1.2** of reviewer 1.

**Comment 2.3:** Line 452 (and other occurrences): The autors discuss the differences in performance of single- and multi-gene models, but it is unclear what we should look for. Please cite the table/figure, and also the R2 value ranges.

**Reply 2.3:** All of the differences discussed in this section refer to the median test *R*^2^ values listed in **Table 2**, we apologize that this was not clear. We now added more references to **Table 2** in this section, make clearer that the models compared are the best multi-gene model (either the best all-gene model or the best TF model) and the best single-gene model, and included $\Delta R^{2}$ ranges for the phenotype sets discussed, with $\Delta R^{2}$ defined as

$\Delta R^{2}=\max\left( {median test R}^{2}\text{ of best all-genes model}, \text{ median test }R^{2} of best TF model \right)- {median test R}^{2}\text{of best single-gene model}$

**Comment 2.4:** The top predictors for leaf and seed phenotypes chapter is somewhat long and tedious to read due to the descriptions of many genes found in the table. Perhaps it would be easier to summarize the findings and phenotypes as another table/figure?

**Reply 2.4:** We agree that the top predictors sections are rather tedious to read, but they contain a lot of information that is relevant to the interpretation of our modeling results. We now tried to make the text more readable. Moving all of the gene descriptions in these sections to a table in the main text was found to be infeasible because of the size of this table, and because the links between the genes and processes found in the top predictor lists could not be represented in a tabular format. The number of genes involved also makes clear figure representations of these links difficult. Moving everything to supplementary is also not an option as this would break the flow of the main text too much and obscure the link between the modeling results and their interpretation. Instead, we now moved all of the ‘details’, e.g. on knockout or overrepresentation phenotypes of the *A. thaliana* orthologs of the top predictor genes, into a new supplementary dataset, **S7 Data**, and shortened the sections in the main text to the extent possible. We hope this has sufficiently improved the readability of these sections.

**Comment 2.5:** Line 429: Is a comparison of the phenotype prediction performance between multi-gene models and transcription factors models relevant?

**Reply 2.5:** This is indeed relevant, apologies for the omission. We’ve now added a section comparing the performance of the all-genes and TF models, essentially concluding that both model types overall have similar performance, in particular for the most predictable phenotypes. This is one of the elements justifying why we focus on the TF models when interpreting the biological relevance of the top leaf and yield phenotype predictors later on in the manuscript.

**Comment 2.6:** Line 455-456: According to the data in Table 2, it seems that most of the shoot dry weight traits are not better predicted by muti-gene models than single-gene models.

**Reply 2.6:** Dr. Mutwil is correct, ‘most’ should have been ‘half’ : $\Delta R^{2}$ = [0.03, 0.04] for the two stem1 dry weight traits and $\Delta R^{2}$ = [0, -0.05] for the two total shoot dry weight traits. In the revised manuscript version, we have downtuned several statements regarding model performance comparisons, in order not to overinterpret small differences in *R*^2^ scores (for which we do not have a significance measure, by lack of an accepted formalism to calculate the significance of out-of-sample *R*^2^ differences, this is work in progress in our group). For instance, where the text in the original version read:

‘For leaf 8 phenotypes, the best multi-gene models generally have only slightly better median test *R*^2^ scores than the best single-gene models (**Table 2**). In other words, multi-gene models offer little benefit over single-gene models for quantitative prediction of leaf 8 phenotypes. For leaf 8 length at 76 DAS and 81 DAS, the best single-gene models even outperform the multi-gene models.’,

we now write:

‘For leaf 8 phenotypes, the best multi-gene models and best single-gene models have comparable median test *R*^2^ scores ($\Delta R^{2}$in the range [-0.05, 0.03], with $\Delta R^{2}=\max\left( {\text{median test} R}^{2}\text{ }\text{of best all-genes model}, \text{ }\text{median test}\text{ }R^{2} \text{of best TF model} \right)- {\text{median test} R}^{2}\text{of best single-gene model}$, **Table 2**). In other words, multi-gene models offer no benefit over single-gene models for quantitative prediction of leaf 8 phenotypes.’

**Comment 2.7:** It would be useful to indicate TFs that are found in RF and enet models if Figure 3, 4.

**Reply 2.7:** We thank Dr. Mutwil for this suggestion, highlighting TFs that are found in multiple models indeed facilitates comparison of the top TF predictor figures. Instead of only highlighting TFs in common between **Fig 3** (RF model for leaf phenotypes) and **Fig 4** (enet model for yield phenotypes), we’ve opted to highlight TFs that are top predictors in either of the leaf phenotype models (**Fig 3** and **S9 Fig**, formerly **Figure S8**) and either of the yield models (**Fig 4** and **S10 Fig**, formerly **Figure S9**) in blue in all figures concerned. Additionally, we’ve highlighted TFs that are found in both the enet and RF models for a given phenotype set (**Fig 3** and **S9 Fig** for leaf phenotypes, or **Fig 4** and **S10 Fig** for yield phenotypes) in red. TFs that are found in both the RF and enet top predictor lists for phenotype set A and are also found in the RF or enet top predictor list for phenotype set B are indicated in magenta (= red + blue).

**Comment 2.8:** Line 495: “Random forest” and “elastic net” abbreviations have been defined in line 330, so no need to define it again in 495. Please also check similar problems throughout the manuscript.

**Reply 2.8:** We removed superfluous definitions of abbreviations, thanks for pointing this out. In the Methods section, we do however prefer to still use full-length words in some instances instead of the associated abbreviations, to improve readability.

**Comment 2.9:** Line 695: Please mark the numbers of TFs in Figure 3 and Figure S8 as well.

**Reply 2.9:** Done.

**Comment 2.10:** Is it possible to further analyze whether top predictors and phenotype are positively or negatively correlated?

**Reply 2.10:** Yes, this is possible. All significant associations (*q* <= 0.05) between genes and phenotypes, as inferred from linear mixed effect models, are listed in **S4 Data** (formerly **Data S4**), we apologize for not referencing this dataset where needed. The coef.x column in **S4 Data** indicates whether a correlation is positive or negative, this is now also explicitly stated in the **S4 Data** legend. In most cases the expression and phenotype profiles shown in **Fig 3**, **Fig 4**, **S9 Fig** (formerly **Figure S8**) and **S10 Fig** (formerly **Figure S9**) are already indicative on whether a given top predictor is positively or negatively correlated with a given phenotype. We did not explicitly discuss all signs of correlations between top predictors and phenotypes in the Results section because in many cases the biological relevance of these correlation signs is difficult to interpret, as elaborated on in the Discussion section for the *B. napus* orthologs of several flowering genes found in the top predictor lists for yield phenotypes, and several leaf development genes found in the top predictor lists for leaf phenotypes. Adding correlation signs everywhere would make the manuscript longer without adding much in terms of content, so we would prefer not to do this.
